# Single-molecule imaging reveals control of parental histone recycling by free histones during DNA replication

**DOI:** 10.1126/sciadv.abc0330

**Published:** 2020-09-18

**Authors:** D. T. Gruszka, S. Xie, H. Kimura, H. Yardimci

**Affiliations:** 1 The Francis Crick Institute, 1 Midland Road, London NW1 1AT, UK.; 2 Cell Biology Center, Institute of Innovative Research, Tokyo Institute of Technology, 4259 Nagatsuta-cho, Midori-ku, Yokohama 226-8503, Japan.

## Abstract

During replication, nucleosomes are disrupted ahead of the replication fork, followed by their reassembly on daughter strands from the pool of recycled parental and new histones. However, because no previous studies have managed to capture the moment that replication forks encounter nucleosomes, the mechanism of recycling has remained unclear. Here, through real-time single-molecule visualization of replication fork progression in *Xenopus* egg extracts, we determine explicitly the outcome of fork collisions with nucleosomes. Most of the parental histones are evicted from the DNA, with histone recycling, nucleosome sliding, and replication fork stalling also occurring but at lower frequencies. Critically, we find that local histone recycling becomes dominant upon depletion of endogenous histones from extracts, revealing that free histone concentration is a key modulator of parental histone dynamics at the replication fork. The mechanistic details revealed by these studies have major implications for our understanding of epigenetic inheritance.

## Teaser

Parental histone dynamics at the replication fork are altered by free histones, leading to controlled eviction or transfer.

## INTRODUCTION

Eukaryotic genomes are organized into chromatin, which influences many cellular processes, ranging from DNA replication and repair to gene transcription. The basic unit of chromatin is a nucleosome, which consists of 145 to 147 base pairs of DNA wrapped around an octameric histone protein core, formed from two copies of each of the four histones: H2A, H2B, H3, and H4. Histones H3 and H4 assemble into a symmetric heterotetramer, and the two H2A–H2B dimers are docked onto the (H3–H4)_2_ tetramer (*1*). Nucleosomes are very stable nucleoprotein complexes, but they are also highly dynamic with regard to their conformation, composition, and positioning within chromatin (*1*, *2*). Nucleosome dynamics control DNA accessibility and are regulated by complex interplay of numerous factors, such as chromatin remodelers, histone chaperones, modifying enzymes, and polymerases (*2*).

Chromatin is partitioned into domains, which either promote (euchromatin) or block (heterochromatin) transcription and hence determine the cellular identity. Nucleosomes in transcriptionally active and silenced chromatin domains carry specific histone posttranslational modifications (PTMs) and/or distinct histone sequence variants (*3*, *4*). Therefore, maintenance of cellular identity through mitotic cell division relies on faithful transfer of information encoded in both DNA sequence (genetic inheritance) and nucleosome landscape (epigenetic inheritance). Semiconservative DNA replication ensures genetic inheritance, but it presents a major challenge to chromatin, which undergoes substantial structural reorganization, starting from disassembly of parental nucleosomes and ending in restoration of nucleosome landscape on daughter strands (*5*, *6*).

To allow parental DNA unwinding and subsequent nascent strand synthesis, each and every nucleosome must be transiently disrupted ahead of the replication fork. Nucleosome destabilization is localized to an average of two nucleosomes immediately ahead of the replication fork and leads to release of the (H3–H4)_2_ tetramer and H2A–H2B dimers from the DNA (*5*, *7*, *8*). It remains unclear what molecular forces trigger the localized nucleosome eviction, but a number of physical and chromatin factors have been implicated in this process, including unzipping and positive supercoiling of the DNA, physical collision between the nucleosome and the replisome, and histone chaperone complex FACT (facilitates chromatin transcription) (*5*, *9*).

After passage of the replication fork, nucleosomes are rapidly reassembled on the two daughter strands, from the pool of recycled parental and newly synthesized histones. Nucleosome reassembly starts with the deposition of (H3–H4)_2_ tetrameric histone core, followed by the association of two H2A–H2B dimers (*5*). Current models suggest that nucleosomes deposited on newly replicated DNA contain either parental or new H3–H4 histones (with the exception of nucleosomes containing H3.3 variant). This implies two distinct replication-coupled nucleosome assembly pathways on nascent DNA: the transfer (recycling) of parental histones released from nucleosomes disrupted by the replisome passage and de novo deposition of newly synthesized histones. Nucleosomal H2A–H2B dimers are more dynamic than (H3–H4)_2_ tetramers and readily exchange with the pool of newly synthesized histones throughout the cell cycle (*5*, *10*). Consequently, old and newly synthesized H2A–H2B dimers can form nucleosomes with both parental and new (H3–H4)_2_ tetramers (*8*, *11*).

Quantitative proteomics studies indicate that nucleosomes deposited on newly replicated DNA are composed of approximately equal amounts of new and old H2A, H2B, H3, and H4 histones (*12*), implying that all parental histones are fully recycled during replication. It has also been reported that parental histones are recycled with PTMs (*12*) and that their genomic localization, whether in active or repressed chromatin, is preserved on daughter strands through histone recycling (*13*–*15*). However, recent studies using mouse embryonic stem cells demonstrated that while repressed chromatin domains are indeed preserved through the local redeposition of parental H3–H4 histones at the replication fork, parental H3–H4 histones associated with active chromatin domains did not exhibit such preservation (*16*). Furthermore, fluorescence imaging–based analysis of parental H3 histone recycling in HeLa cells over two cell divisions revealed rates of parental histone loss that were higher than the expected 50% per cell cycle (*17*).

Recent studies into the mechanism of parental histone segregation onto replicating DNA showed that histones H3–H4 distribute more or less equally between the two strands (*18*, *19*). Several replisome components are involved in parental histone segregation. The N-terminal domain of MCM2 (minichromosome maintenance complex component 2), a component of the CMG replicative helicase, contains a histone H3–H4-binding domain and promotes the transfer of parental H3–H4 to the lagging strand (*18*), in association with the Ctf4 (chromosome transmission fidelity 4) adaptor protein and polymerase alpha (Pol α) (*20*). Dpb3 and Dpb4, two nonessential subunits of yeast polymerase epsilon (Pol ε), facilitate the parental H3–H4 transfer to the leading strand (*19*). In addition, various histone chaperones, chromatin factors, and other replisome components have been implicated in parental histone recycling and/or the inheritance of chromatin states (*5*, *6*, *9*). Together, these findings support a mechanism for replication-coupled parental histone recycling whereby, upon eviction from the DNA, parental histones H3–H4 are retained close to the replisome through a series of protein-protein interactions, resulting in their targeted and localized redeposition behind the replication fork. Alternative models propose passive histone transfer, supported by DNA loop formation (*6*, *21*, *22*).

To date, most studies looking into the mechanisms of replication-coupled parental histone recycling use bulk and/or steady-state approaches, which lack the spatial and temporal resolution needed to unravel the molecular detail of this highly dynamic process. Here, we report a real-time single-molecule imaging platform that uses microfluidics-based DNA replication in *Xenopus laevis* egg extracts, protein engineering, and total internal reflection fluorescence (TIRF) microscopy to gain insight into the outcome of replication fork collision with nucleosomes. Our approach allows simultaneous visualization of parental histones and replication forks as they navigate through the nucleosomal environment of individual DNA molecules.

## RESULTS

### Fluorescent λ nucleosomes are discretely distributed as “beads on a string”

**Fig. 1:**
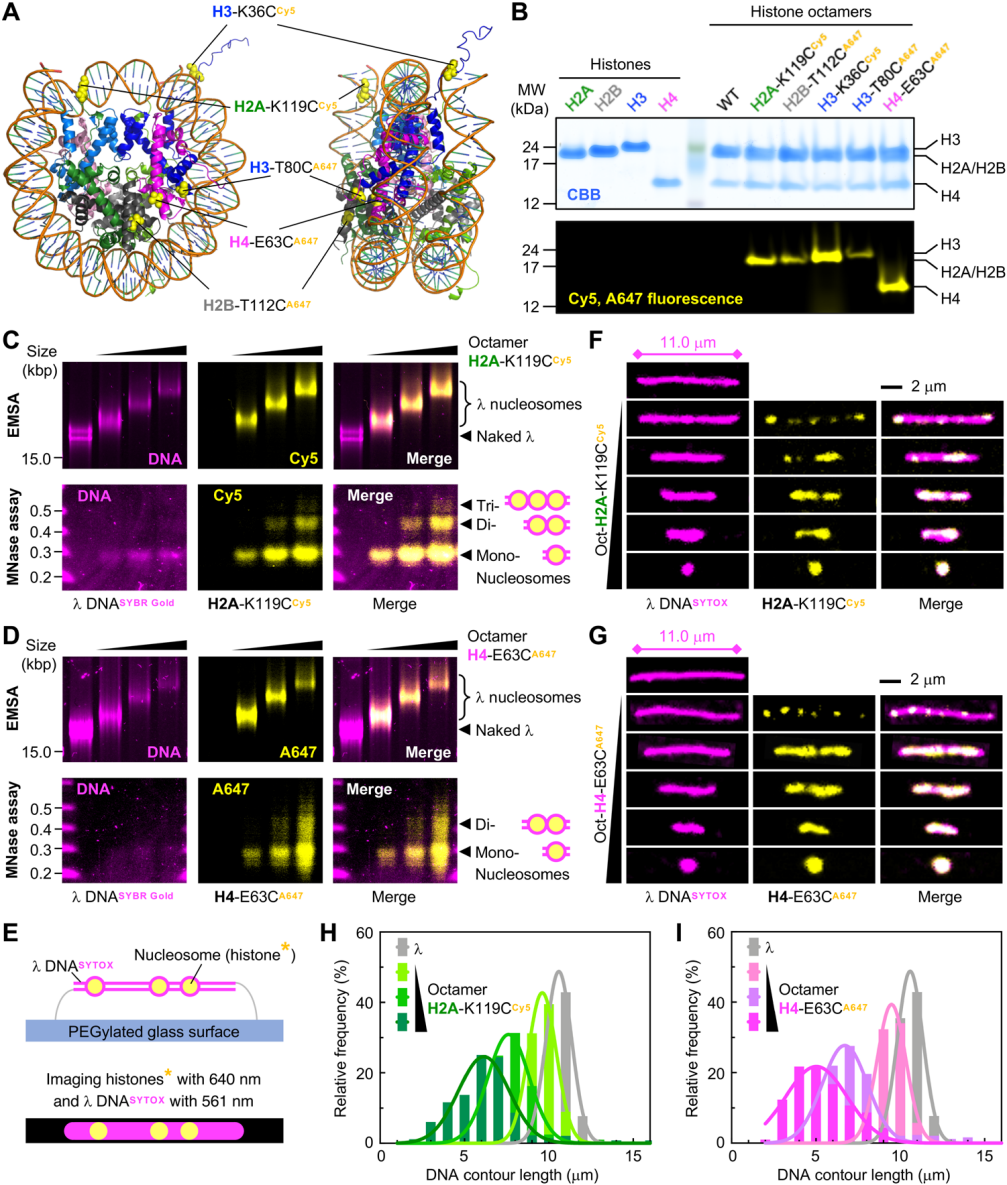
Fluorescent nucleosomes on λ DNA are discretely distributed in a beads-on-a-string manner. (**A**) Crystal structure of the *Xenopus* nucleosome (Protein Data Bank 1AOI) illustrating the location and type of fluorescent dye (Cy5 or Alexa Fluor 647, abbreviated as A647) used to label histones. (**B**) SDS–polyacrylamide gel electrophoresis analysis of wild-type (WT) and fluorescently labeled histones and histone octamers. MW, molecular weight. (**C** and **D**) Native EMSA (top) and MNase protection assay (bottom) for nucleosomes labeled at H2A-K119C^Cy5^ (C) and H4-E63C^A647^ (D) reconstituted on λ DNA at increasing DNA:octamer ratios (1:0, 1:40, 1:120, and 1:200). kbp, kilobase pair. (**E**) Schematic of fluorescent λ nucleosomes immobilized in the microfluidic device for single-molecule imaging. (**F** and **G**) Single-molecule imaging of nucleosomes labeled at H2A-K119C^Cy5^ (F) and H4-E63C^A647^ (G) reconstituted on λ DNA at increasing DNA:octamer ratios (1:0, 1:50, 1:125, 1:200, 1:275, and 1:350). (**H** and **I**) Single-molecule quantification of the DNA contour length for nucleosomes labeled at H2A-K119C^Cy5^ (H) and H4-E63C^A647^ (I) reconstituted on λ DNA at increasing DNA:octamer ratios (1:0, 1:40, 1:120, and 1:200). The DNA length of individual molecules was measured on the basis of SYTOX Orange staining of the DNA (approximately 400 molecules at each histone octamer concentration).

Building on the single-molecule methodology developed by Loveland *et al.* (*23*), which allows real-time imaging of growing replication bubbles in *Xenopus* egg extracts, we aimed to visualize fork collisions with nucleosomes. *X. laevis* histones were expressed and purified from *Escherichia coli* and then labeled with small fluorescent dyes. To ensure that our observations are representative of nucleosome dynamics, we labeled all four histones, at various positions in their structure, using different fluorophores (Fig. 1, A and B). Histone octamers, containing one of the four histones labeled fluorescently, were then used to reconstitute nucleosomes on biotinylated λ DNA by NaCl gradient dialysis. By varying the octamer:DNA molar ratio in our reconstitution reactions, we achieved different levels of nucleosome saturation. Histone deposition and correct nucleosome folding on λ DNA were verified by electrophoretic mobility shift assay (EMSA) and micrococcal nuclease (MNase) protection assay (Fig. 1, C and D, and fig. S1).

To visualize fluorescent nucleosomes on individual λ DNA molecules, we used microfluidics devices with a polyethylene glycolylated (PEGylated) and streptavidin-functionalized glass surface in combination with TIRF microscopy. Biotinylated λ DNA molecules containing fluorescently labeled nucleosomes were first stretched under flow (to approximately 70% of the maximally stretched form) and tethered at both ends to the surface (Fig. 1E). The fluorescent DNA stain SYTOX Orange was then introduced into the chamber, and both the DNA and fluorescent histone in nucleosomes were imaged. Fluorescent nucleosomes are discretely distributed on SYTOX-stained λ DNA as “beads on a string” (Fig. 1, F and G). As more nucleosomes are deposited on the DNA, the associated fluorescence signal of the labeled histone increases and appears more contiguous. Consistent with the DNA wrapping around the octameric histone core, we also observed apparent shortening of the λ DNA contour length upon nucleosome deposition, in a nucleosome density (histone octamer concentration)–dependent manner. We quantified this “shortening” effect for samples shown in Fig. 1 (C and D) by measuring the contour length of approximately 400 individual molecules per sample and plotting the histograms (Fig. 1, H and I). At very high histone octamer:DNA ratios, the molecules appeared as intense diffraction-limited spots of fluorescence (Fig. 1, F and G, bottom) that, in contrast to singly tethered low-density nucleosomal templates, did not stretch under buffer flow (movies S1 and S2).

### Assay for single-molecule imaging of parental histones during DNA replication

**Fig. 2:**
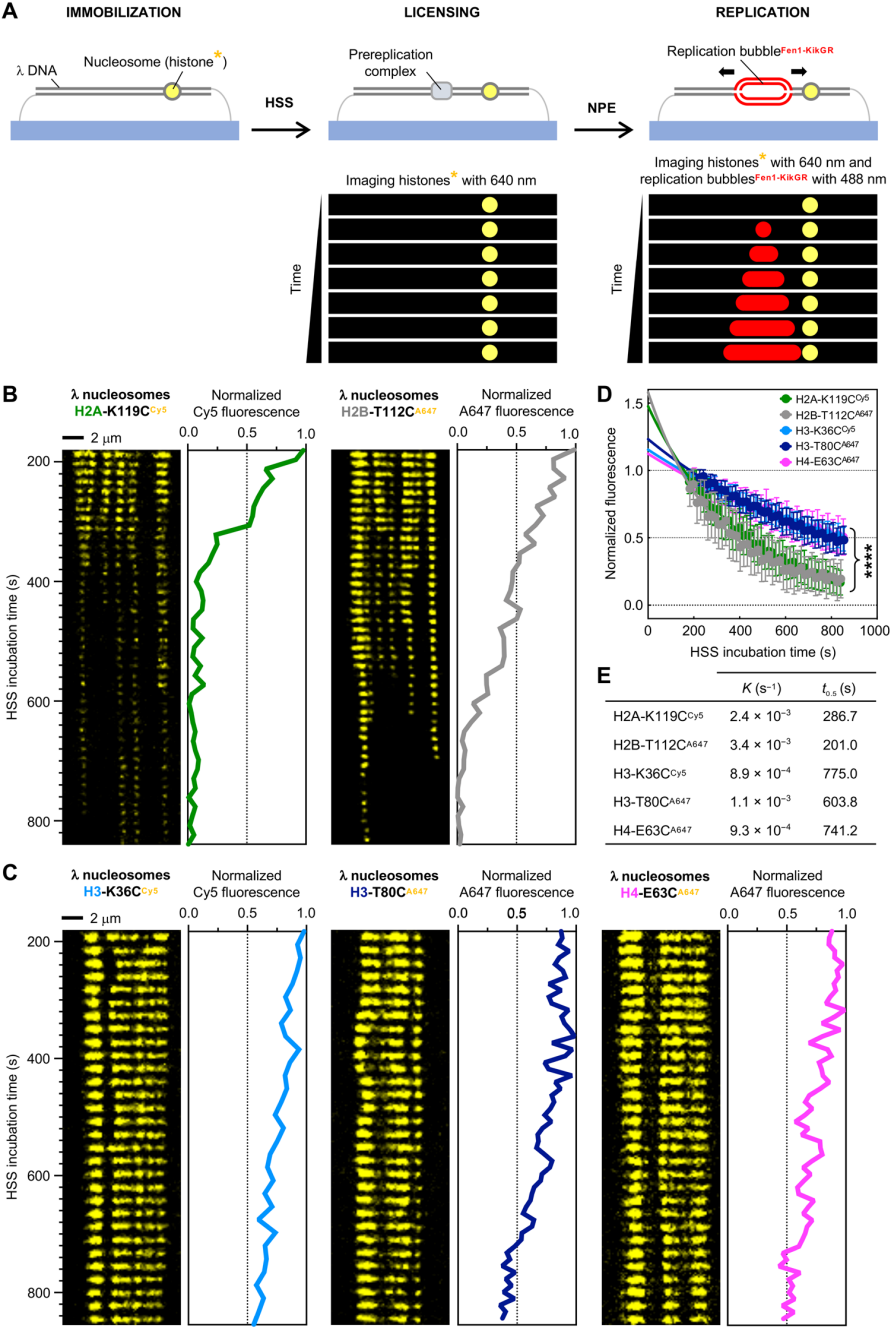
Histone dynamics during DNA licensing in HSS. (**A**) Schematic of the experimental setup for real-time single-molecule imaging of nucleosome dynamics during replication in *X. leavis* egg extracts. The immobilized DNA is licensed in high-speed supernatant (HSS). Bidirectional replication is initiated upon introduction of nucleoplasmic extract (NPE) supplemented with a fluorescent fusion protein Fen1-KikGR, which decorates replication bubbles. (**B** and **C**) Kymograms and corresponding intensity profiles for fluorescent λ nucleosomes during incubation in HSS. Nucleosomes labeled at H2A-K119C^Cy5^ and H2B-T112C^A647^ (B) show faster loss of fluorescence than nucleosomes labeled at H3-K36C^Cy5^, H3-T80C^A647^, and H4-E63C^A647^ (C). (**D**) Plot showing the mean loss of fluorescent signal for λ nucleosomes during incubation in HSS. More than 100 molecules were analyzed for each histone template. Individual fluorescence decay traces were normalized to background (“0”) and maximum value of fluorescence (“1”). A mean fluorescence value and SD were calculated and plotted for each time point. The mean value traces were then fitted to a single exponential function. (**E**) Summary of the fluorescence decay rate constants (*K*) and half-lives (*t*_0.5_) extracted from the single exponential fit to the data presented in (D). See table S1 for detailed fitting parameters.

To investigate the dynamics of parental histones during DNA replication, we combined real-time TIRF imaging with microfluidics-based replication assays in nucleus-free *X. laevis* egg extracts (Fig. 2A) (*23*–*25*). Surface-immobilized, stretched λ nucleosomes were incubated in a high-speed supernatant (HSS) of *Xenopus* eggs to promote sequence nonspecific origin licensing (i.e., the origin recognition complex–dependent assembly of prereplication complexes). Next, a concentrated nucleoplasmic extract (NPE) was introduced into the microfluidic chamber, which initiates and supports efficient bidirectional replication. The number of replication initiations per DNA template was regulated by adding the Cdk2 inhibitor p27^Kip^ (*26*). To allow visualization of replication fork progression in real time, NPE was supplemented with Fen1-KikGR, a *Xenopus* flap endonuclease 1 (Fen1; binds to PCNA and displaced 5′ flap of the Okazaki fragments) fused to monomeric photoactivatable protein KikGR. Fen1-KikGR decorates replication bubbles but does not detectably alter ensemble replication kinetics or replication bubble growth monitored in single-molecule assays (*23*).

### Differential exchange kinetics of H2A/H2B versus H3/H4 during DNA licensing

We first investigated histone dynamics during DNA licensing (Fig. 2, B to E, and movies S3 to S7). As the extract reached immobilized λ nucleosomes, thermal fluctuations of individual molecules became gradually reduced in comparison to buffer conditions, because of the DNA being bound by extract proteins, including native histones (see Materials and Methods and Supplementary Text for further details). We found that all analyzed fluorescent histones (Fig. 2, B and C) show limited lateral dynamics, i.e., their movement along the DNA molecule is largely confined within the spatiotemporal resolution of our approach. While there was some local drift of histone fluorescence from the starting position, we never observed long-distance lateral movement for any of the histones tested in our assay. The dominant dynamic behavior observed during HSS incubation is the gradual loss of histone-associated fluorescence over time, occurring at a significantly faster rate than the measured rate of photobleaching in buffer (fig. S2). In the case of H2A-K119^Cy5^ and H2B-T112C^A647^ (Fig. 2B and movies S3 and S4), the decrease in histone fluorescence signal was greater than for H3-K36C^Cy5^, H3-T80C^A647^, and H4-E63C^A647^ (Fig. 2C and movies S5 to S7). We quantified the average rate of histone fluorescence decay for each template (Fig. 2, D and E, and table S1) and found that histones H3 and H4 have approximately three times longer half-lives in HSS than H2A and H2B.

The observed loss of histone fluorescence is likely to result from three phenomena: (i) photobleaching of the dye, (ii) nucleosome eviction, and (iii) fluorescent histone exchange within a nucleosome with an unlabeled native counterpart from the extract. Given that the same type of dye was used to track histones displaying different kinetic behavior (e.g., H2B-T112C^A647^ and H4-E63C^A647^; half-lives, 201.0 and 741.2 s^−1^, respectively) and that histone H3 labeled with two different dyes showed a similar rate of fluorescence decay (H3-K36C^Cy5^ and H3-T80C^A647^; half-lives: 775.0 and 603.8 s^−1^, respectively), we conclude that photobleaching itself cannot account for the observed kinetic differences between H2A/H2B and H3/H4. We also rationalized that nucleosome eviction would affect the fluorescence signal in the same way, regardless of the histone type, as all four histones would simultaneously dissociate from the DNA. Hence, we conclude that the observed difference in the loss of fluorescence between H2A/H2B and H3/H4 predominantly reflects different exchange rates with native histones, present in HSS at a concentration of ~1 to 6 μM (fig. S3A). The faster displacement rate for histones H2A and H2B relative to H3 and H4 in *Xenopus* extracts is consistent with previous reports indicating greater lability of H2A-H2B within nucleosomes in vivo (*5*, *7*, *10*) and could potentially reflect the structural organization of the histone octamer, where the two H2A-H2B dimers are more accessible than the core (H3–H4)_2_ heterotetramer (Fig. 1A).

### Replication of fluorescent nucleosomal templates

To investigate the dynamics of parental histones during DNA replication, we initiated replication of the stretched and licensed fluorescent λ nucleosomes by introducing NPE containing Fen1-KikGR (Fig. 2A). After one or two origins per template had fired, the NPE mix was replaced with NPE supplemented with p27^Kip^ to prevent further origin firing. This procedure allowed us to follow the growth of individual replication bubbles in real time and hence to determine the outcome of collision between a single progressing replication fork with nucleosomes on its path. We anticipated that as long as fluorescent nucleosomes are sparsely distributed along the stretched DNA molecules (i.e., a few fluorescent nucleosomes per DNA molecule), we would be able to distinguish individual fork-nucleosome collision events.

We first examined whether replication of nucleosomal λ templates is as efficient as that of naked λ. To this end, we measured the mean replication fork velocity for naked λ, as well as wild-type and fluorescent nucleosomes on λ DNA. For all templates, the replication forks traveled at a similar mean velocity of approximately 640 nt/min (fig. S3, B and C), indicating that neither the reconstituted nucleosomes nor the fluorophores that they carry affect fork progression. Forks fired on similar time scales for naked DNA and low-density nucleosomal templates, between 5 and 12 min from the moment of NPE introduction. In addition, we compared the efficiency of replication using ensemble assays, in which naked plasmid and plasmid containing fluorescent nucleosomes were replicated under unrestricted firing conditions in *Xenopus* egg extracts. We found that chromatinized plasmids replicated as efficiently as their naked counterpart (fig. S3D). We conclude that fluorescent λ nucleosomes and microfluidics-based replication assays provide an appropriate imaging platform for tracking the fate of parental histones during replication.

### Heterogeneous dynamics of parental histones upon replication fork arrival

**Fig. 3:**
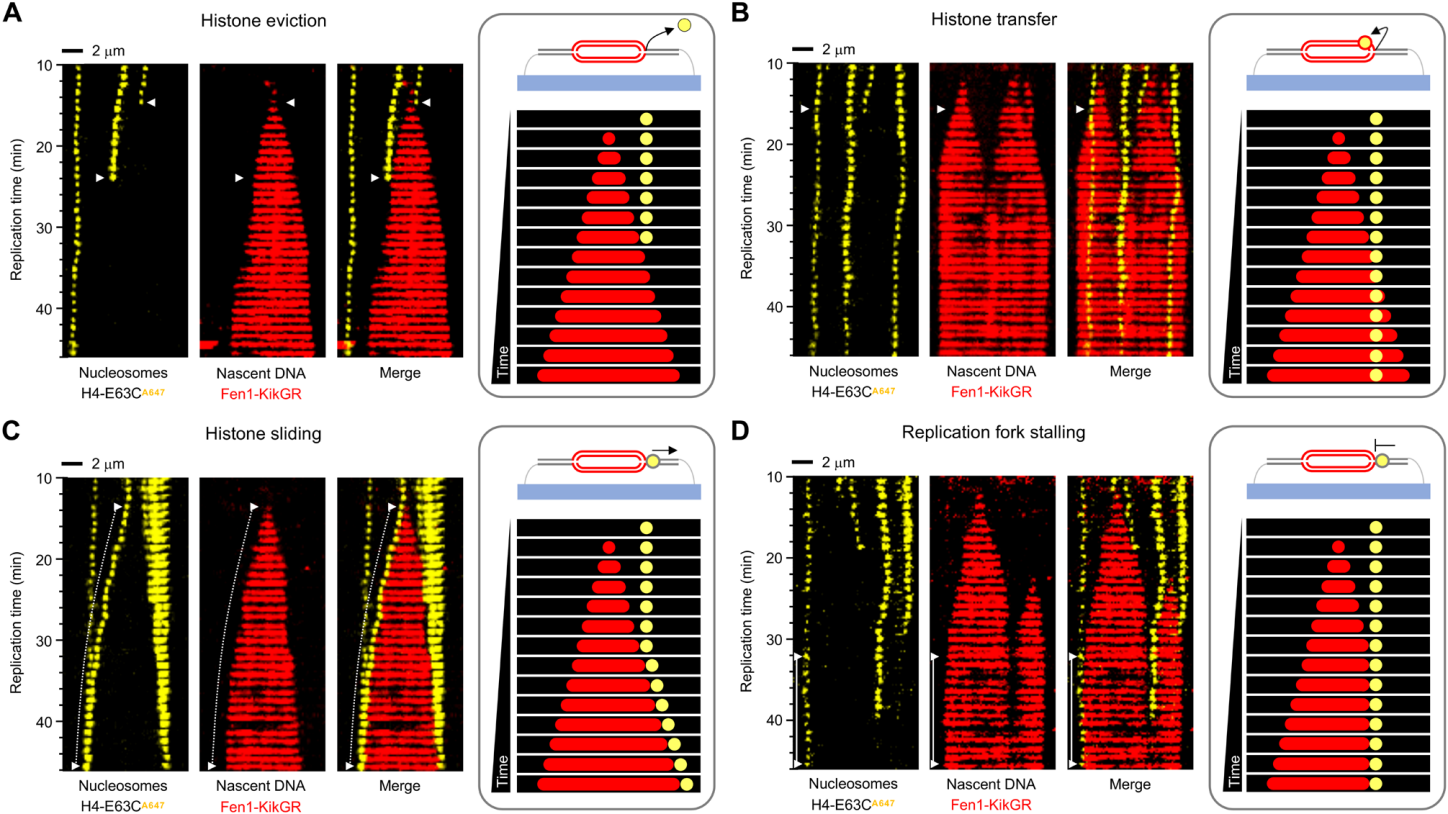
Heterogeneous dynamics of parental histones upon replication fork arrival. For each specified outcome, data are presented as kymograms of nucleosome-associated fluorescence (H4-E63C^A647^; yellow, left), Fen1-KikGR signal indicating nascent DNA (red, middle) and both signals together (merge, right). Time and size scales are presented. The white triangles mark the point of initial encounter between the replication fork and nucleosome. Dotted lines indicate sliding events, whereas solid lines correspond to replication fork stalling. For clarity, a schematic representation of each outcome is shown in gray borders. (**A**) Histone eviction is manifested by the loss of histone fluorescence at the point of collision with the replication fork. (**B**) Histone transfer is observed when the histone-associated fluorescence is retained and incorporated into the track of replicated DNA. (**C**) Histone sliding is observed when the histone-associated fluorescence moves together with the tip of the replication bubble (marked as a dotted white line). (**D**) Replication fork stalling occurs when nucleosome constitutes a roadblock preventing the replication fork from further movement. It is manifested in the kymogram as an arrested tip of the replication bubble next to a static histone signal (indicated as a solid line).

To determine the outcome of replication fork encounters with parental nucleosomes, we focused on low–nucleosome density λ templates containing either H3-K36C^Cy5^ or H4-E63C^A647^, because of their high fluorophore labeling efficiency and lower exchange rates, compared to H2A-H2B, during licensing in HSS. We observed four basic outcomes of replication fork collision with nucleosomes: histone eviction, localized parental histone transfer onto daughter strands, histone sliding ahead of the replication fork, and replication fork stalling (Fig. 3 and movies S8 to S11).

Histone eviction is emblematic of nucleosome disassembly before DNA unwinding and synthesis, resulting in parental histone release into the pool of free histones. It is manifested in the kymograms and accompanying movies by the loss of histone fluorescence at the point of encounter with the replication fork (Fig. 3A and movie S8). In the case of histone transfer, the histone-associated fluorescence is incorporated into the Fen1-KikGR–decorated track of nascent DNA upon passage of the replication fork (Fig. 3B and movie S9). This characteristic illustrates localized parental histone recycling, a mechanism whereby the fluorescent histone from disassembled parental nucleosome stays in the vicinity of the replisome and is immediately redeposited into a nucleosome on daughter DNA. The resolution of our technique is approximately 1 kilobase pair (kbp), and so it does not allow us to specify if histones are reinstated at the exact same locus within the replicated DNA. It is important to note that our data are consistent with both active (replisome-assisted) and passive (DNA loop–mediated) mechanisms proposed for parental histone transfer behind the replication fork (*6*, *21*, *22*). The third type of event, which we classify as histone sliding, is detected as a continuous movement of histone fluorescence signal with a tip of the replication bubble from the moment of nucleosome-fork encounter (Fig. 3C and movie S10). This sliding behavior is likely indicative of two molecular phenomena, which, at present, cannot be distinguished. One possibility is that the whole nucleosome is being pushed ahead of the replication fork, as observed for nucleosome remodelers (*27*). Alternatively, the nucleosome is disassembled at the point of fork collision; the fluorescent histone then associates with the replisome and travels with it along the DNA. Sliding typically occurs over short distances (within a few kbp), but occasionally, we observed histone push on a scale of 25 to 30 kbp, spanning over a half of the length of λ DNA (48.5 kbp). Replication fork stalling upon collision with a nucleosome is exemplified in our experiments by a static histone fluorescence next to an arrested tip of the replication bubble (Fig. 3D and movie S11). In this scenario, the nucleosome acts as a roadblock preventing the replication fork from further movement.

**Fig. 4:**
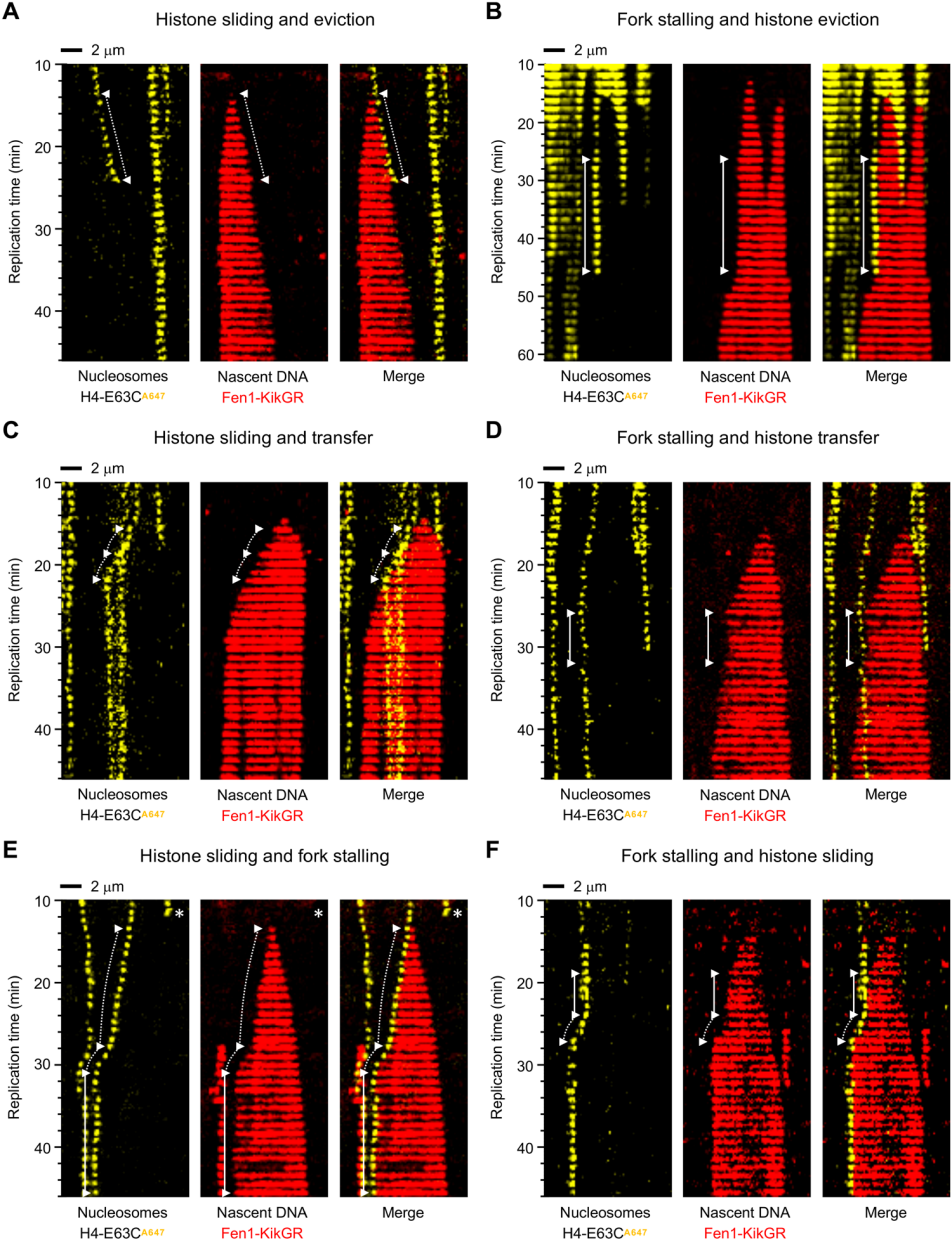
Secondary outcomes of the replication fork collision with nucleosomes during DNA replication in *Xenopus* egg extracts. For each specified outcome, data are presented as kymograms of nucleosome-associated fluorescence (yellow, left), Fen1-KikGR signal indicating nascent DNA (red, middle) and both signals together (merge, right). Time and size scales are presented. The white triangles mark the point of initial encounter between the replication fork and nucleosome. Dotted lines indicate sliding events, whereas solid lines correspond to replication fork stalling. Replication-independent histone loss is marked in (E) with a white asterisk. (**A**, **C**, and **E**) Histone sliding can terminate in histone eviction (A), histone transfer (C), and replication fork stalling (E). (**B**, **D**, and **F**) Replication fork stalling can lead to histone eviction (B), histone transfer (D), and histone sliding (F).

Nucleosome eviction and localized histone transfer are the two ultimate outcomes of replication fork encounter with nucleosomes as, once they have occurred, the fork and histone are no longer in contact/proximity. In contrast, histone sliding and replication fork stalling preserve the fork-histone “interaction” and hence often lead to secondary outcomes (Fig. 4). Both sliding and stalling can terminate in histone eviction (Fig. 4, A and B, and movies S12 and S13, respectively) as well as histone transfer behind the replication fork (Fig. 4, C and D, and movies S14 and S15, respectively). In addition, histone sliding can result in replication fork stalling (Fig. 4E and movie S16) and vice versa (Fig. 4F and movie S17). Occasionally, we observe tertiary events; for example, fork stalling followed by histone sliding leads to a second fork stalling (Fig. 4F; note that the second fork stalling event is unmarked) or histone sliding followed by fork stalling terminates in histone transfer (fig. S6A, histone sliding; note that fork stalling and histone transfer are unmarked).

### Histone eviction is the dominant outcome of fork encounter with nucleosomes

**Fig. 5:**
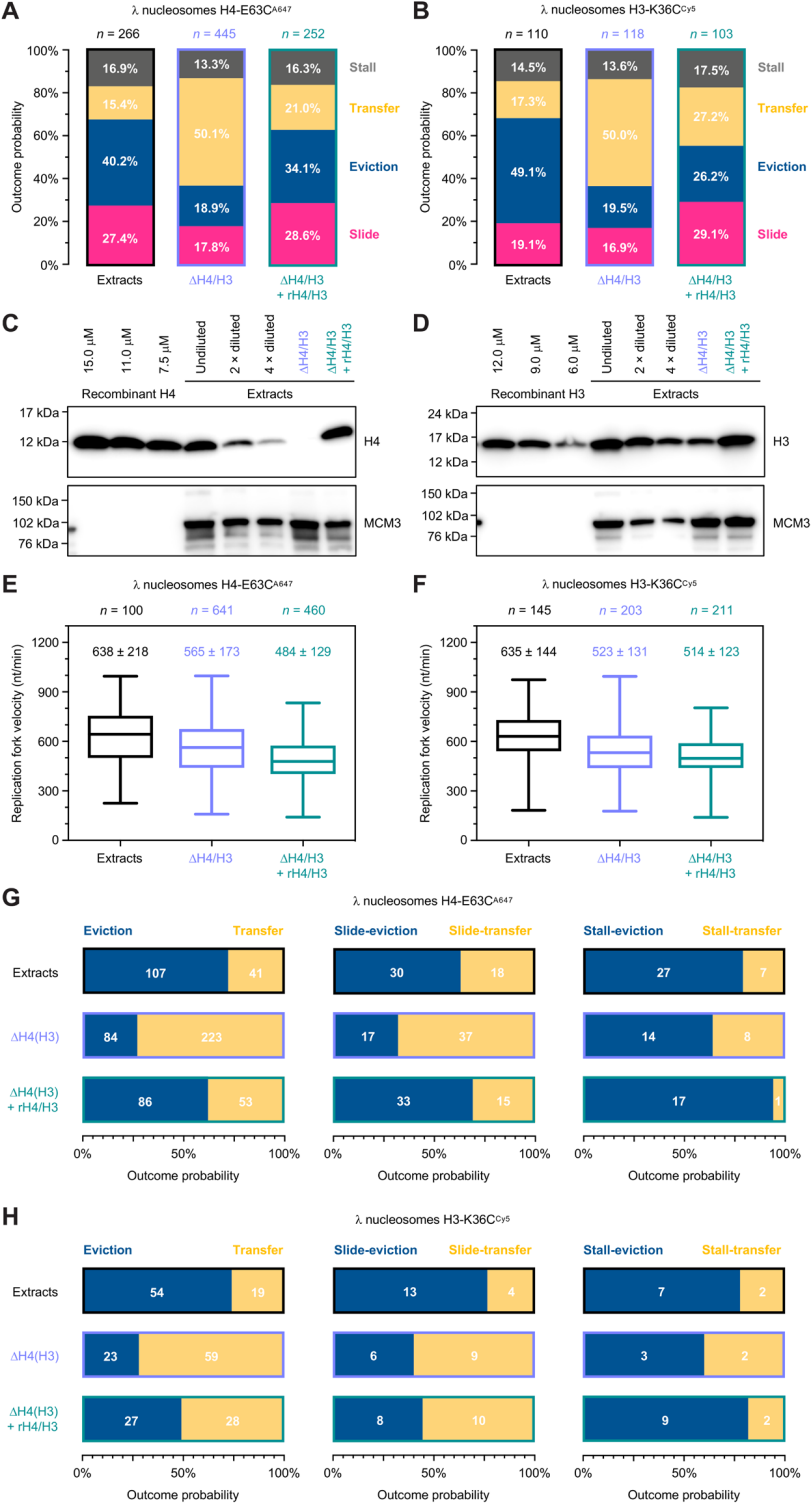
Effect of free histones on parental histone dynamics at the replication fork. (**A** and **B**) Quantification of the four basic outcomes of replication fork collision with nucleosomes labeled at H4-E63C^A647^ (A) and H3-K36C^Cy5^ (B) in regular extracts, extracts depleted of histone H4 and H3 (ΔH4/H3), and depleted extracts supplemented with recombinant histones H4 and H3 (ΔH4/H3 + rH4/H3). *n* indicates the total number of analyzed collisions. All fork-nucleosomes collisions observed during the 60-min replication reaction were analyzed. Data from at least two biological repeats were pooled in the analysis for each tested condition. (**C** and **D**) Western blots used to estimate the concentration of histone H4 (**C**) and H3 (**D**) in extracts. (**E** and **F**) Tukey plot of replication fork velocities measured in extracts for λ nucleosomes containing H4-E63C^A647^ (E) and H3-K36C^Cy5^ (F). Values above the box plots indicate the mean replication fork velocity extracted from the Gaussian fit (±SD). The number of values analyzed per dataset (*n*) is also shown. (**G** and **H**) Quantification of histone eviction versus transfer for nucleosomes labeled at H4-E63C^A647^ (G) and H3-K36C^Cy5^ (H). Analysis for primary (eviction versus transfer) and secondary (slide/stall-eviction versus slide/stall-transfer) outcomes is presented.

To gain further insight into the mechanism of chromatin replication, we quantified the probability of different outcomes of fork-nucleosome encounter in *Xenopus* egg extracts (Fig. 5, A and B, left). Contrary to our expectations, for both tested nucleosomal templates, containing either H4-E63C^A647^ or H3-K36C^Cy5^, the dominant event was histone eviction at 40.2 and 49.1%, respectively. In addition to the replication fork–associated histone evictions, we also observed histone loss independent of replication (Fig. 4E and Supplementary Text). Note that replication fork–independent events were not included in the statistical analysis of collision outcomes. Parental histone recycling at the replication fork, the event that we anticipated to be the most frequent, occurred at significantly lower frequency, 15.4% for H4-E63C^A647^ nucleosomes (the rarest event of all) and 17.3% in the case of nucleosomes carrying H3-K36C^Cy5^. Histone sliding was more prevalent on templates with H4-E63C^A647^ nucleosomes (27.4%) than H3-K36C^Cy5^ (19.1%); however, in both cases, it represented the second most probable outcome of replication fork collision with nucleosomes. Replication forks stalled on nucleosomes in 16.9% of cases for H4-E63C^A647^ and 14.5% for H3-K36C^Cy5^.

In the light of this unexpected inefficient parental histone recycling at the replication fork, we next wanted to test whether DNA stretching could influence the outcome of nucleosome-fork collision. It is plausible that, if a specific three-dimensional fork-replisome structure is needed for efficient parental histone transfer onto daughter strands, the double-tethering of nucleosomal templates (stretched to ~70% of the maximum contour length) could potentially impede longer-range DNA contacts and histone recycling. To address this issue, we performed single-molecule replication experiments, in which approximately 50% of the immobilized nucleosomal λ molecules were tethered to the surface at only one end and so were free to fold in three-dimensional space, unlike their doubly tethered counterparts (fig. S4). Singly tethered molecules in extracts appear as a diffraction-limited spot of fluorescence, which does not stretch under flow of native buffers (because of DNA compaction caused by extract protein binding; see also Supplementary Text), making it impossible to visualize individual fork collisions with nucleosomes. Hence, we compared the loss of histone-associated fluorescence between singly and doubly tethered nucleosomal templates as a proxy for determining the effect of DNA stretching on parental histone retention during replication. We found no difference in the rate of histone loss or daughter strand synthesis (as measured by the increase of the Fen1-KikGR signal) between the two templates (fig. S4, D and E). On the basis of these observations, we conclude that DNA stretching does not cause excessive histone eviction during replication in *Xenopus* egg extracts.

Other potential causes of inefficient histone recycling at the replication fork in our system could be the sparse distribution of fluorescent nucleosomes on stretched λ or the retention of Fen1-KikGR on nascent DNA. To test these scenarios, we conducted single-molecule replication experiments on doubly tethered λ molecules containing higher density fluorescent nucleosomes in extracts supplemented with either Fen1-KikGR or digoxigenin–deoxyuridine triphosphate (dig-dUTP) (fig. S5). The real-time Fen1-KikGR–supplemented experiment clearly demonstrated that most H3-K36C^Cy5^–labeled histones are evicted from the DNA template upon the replication fork arrival (fig. S5A), indicating that nucleosome density does not influence the efficiency of parental histone recycling. Incorporation of dig-dUTP into nascent DNA does not allow us to track the growth of replication bubbles in real time, but it enables their postreplication visualization through immunostaining with fluorescein-labeled anti-digoxigenin antibody (anti-dig Ab^Fluor^). We combined three modes of detection after replication (nucleosomes, H3-K36C^Cy5^; nascent DNA, anti-dig Ab^Fluor^; all DNA, SYTOX Orange) and found that the replicated tracts of λ DNA were largely free of H3-K36C^Cy5^ signal, whereas the nonreplicated λ regions remained decorated with H3-K36C^Cy5^-nucleosomes (fig. S5B). These results further confirm that histone recycling is highly inefficient during replication in *Xenopus* egg extracts and lead us to conclude that Fen1-KikGR does not interfere with histone transfer onto daughter strands.

### Efficiency of histone recycling depends on the concentration of free histones

In *X. leavis* embryos, transcription is activated in the 13th cell cycle (*28*). Until this point, the embryonic genome is transcriptionally silent, and so the oocyte must provide histones in sufficient abundance to support the initial 12 rounds of replication after fertilization. *Xenopus* egg extracts must therefore contain a high proportion of free histones, at least 2^12^ times higher than an equivalent extract of somatic cells. Thus, we set out to determine whether the probabilities of the four outcomes of fork-nucleosome encounter would be different in extracts containing less histones.

We estimated the concentration of histones in our replication-promoting extracts by Western blots as approximately 10 and 20 μM for H4 and H3, respectively (Fig. 5, C and D). Newly synthesized histone H4 is acetylated at Lys12 (H4-K12ac) and forms a predeposition complex with histone H3 (*29*). We depleted extracts of histone H4, using an antibody recognizing H4-K12ac (*30*), to less than 10% of its normal content; estimated concentration of H4 in depleted extracts is ~1 μM (Fig. 5C). This procedure also led to codepletion of histone H3 from extracts and reduced its concentration to ~5 μM (equivalent to 25% of its normal content; Fig. 5D). We next performed single-molecule replication assays on doubly tethered λ nucleosomes, containing either H4-E63C^A647^ or H3-K36C^Cy5^, in extracts depleted of histones H4 and H3. For both templates, we observed a slight reduction in the mean replication fork velocity relative to regular extracts (565 nt/min from 638 nt/min for H4-E63C^A647^ and 523 nt/min from 635 nt/min for H3-K36C^Cy5^; Fig. 5, E and F). The four principal outcomes of fork-nucleosome encounter were still detected in depleted extracts (fig. S6A), but the probability of collision outcomes was different (Fig. 5, A and B, middle), in particular, regarding parental histone transfer and eviction (Fig. 5, G and H). In stark contrast to regular extracts, the dominant event in histone-depleted extracts was localized histone transfer, detected in 50% of collisions for both H4-E63C^A647^ and H3-K36C^Cy5^ λ nucleosomes. This increased efficiency of histone recycling was accompanied by a marked drop in the frequency of histone eviction (18.9% for H4-E63C^A647^ and 19.5% for H3-K36C^Cy5^), whereas histone sliding and replication fork stalling were observed at similar probability levels to those found in undepleted extracts. We also observed a higher probability of secondary transfer events (i.e., slide-transfer and stall-transfer), when compared to regular extracts (Fig. 5, G and H, and fig. S7).

Given the lower mean replication fork velocity in extracts depleted of histones H4 and H3, we next investigated whether the observed increase in localized histone transfer is due to slower replication forks. If that was the case, in regular undepleted extracts, the mean velocity of forks leading to histone transfer upon collision with nucleosomes would be lower than for forks prompting histone eviction. We compared replication fork velocities leading to different outcomes upon nucleosome-fork encounter in regular extracts and detected no such difference (fig. S8). We found no correlation between replication fork speed and any of the nucleosomal outcomes evident during replication in extracts.

Our results strongly suggest that excess provision of free histones during replication, as found in *Xenopus* egg extracts, leads to impaired localized histone recycling. To ensure that the observed effect is specific to histone depletion, we supplemented depleted extracts with recombinant histones H4 and H3 to native concentrations (Fig. 5, C and D) and performed single-molecule replication assays. If our model is correct, then the presence of recombinant histones should counteract the H4/H3 depletion effect and mimic the behavior of regular undepleted extracts. We replicated nucleosomal templates containing either H4-E63C^A647^ or H3-K36C^Cy5^ and detected a slight reduction in the mean replication fork velocity, in comparison to depleted extracts (484 nt/min from 565 nt/min for H4-E63C^A647^ and 514 nt/min from 523 nt/min for H3-K36C^Cy5^; Fig. 5, E and F). Next, we quantified the probability of different fork-nucleosome encounter outcomes in depleted extracts supplemented with recombinant histones (Fig. 5, A and B, right, and fig. S6B). Consistent with our predictions, we found reduced levels of histone transfer (21.0% for H4-E63C^A647^ and 27.2% for H3-K36C^Cy5^) and higher-frequency eviction events (34.1% for H4-E63C^A647^ and 26.2% for H3-K36C^Cy5^), relative to histone-depleted extracts (Fig. 5, A, B, G, and H). A similar trend was also observed for secondary transfer and eviction events (Fig. 5, G and H, and fig. S7), i.e., events following initial slide and stall. In the case of H4-E63C^A647^ λ nucleosomes, histone sliding and replication fork stalling were detected at similar probability levels to those found in regular and undepleted extracts (Fig. 5, A and G). We note that for λ nucleosomes containing H3-K36C^Cy5^ (Fig. 5, B and H), these two events were found at a slightly higher frequency than previously detected for regular and undepleted extracts. On the basis of these data, we conclude that the efficiency of localized histone recycling at the replication fork depends on the concentration of soluble histones.

## DISCUSSION

Chromatin domains and their constituent histones with specific PTMs define the transcriptional program of the cell and hence must be faithfully replicated through cell division. During replication, chromatin undergoes a complete nucleosome-by-nucleosome disassembly, followed by restoration of chromatin structures on the daughter strands. Because of the dynamic and multicomponent nature of chromatin replication, the molecular mechanisms that govern nucleosome disassembly and parental histone transfer remain poorly characterized. In this work, we devised a real-time single-molecule imaging platform to determine the fate of parental nucleosomes and their constituent histones upon encounter with progressing replication forks. Our approach enables visualization of individual nucleosome-fork collisions during replication in *Xenopus* egg extracts and thus allowed us to monitor chromatin replication at an unprecedented spatiotemporal resolution. Broader implications and significance of our findings are discussed below.

### Implications of heterogeneous histone dynamics upon collision with the fork

The current consensus model for replication-coupled parental histone transfer suggests that (i) most, if not all, parental histones are recycled at the replication fork (*12*), (ii) parental histones are quickly deposited onto nascent DNA and are equally distributed between the leading and lagging strands (*10*, *11*, *18*, *19*), (iii) parental histones are recycled with their specific PTMs (*12*), and (iv) that genomic localization of parental histones is preserved on daughter strands (*13*). Most of these pioneering studies are based on tailored chromatin immunoprecipitation sequencing and proteomics approaches that, while yielding important insights into replication-coupled chromatin restoration in bulk, inevitably, average out any inhomogeneities. They also do not provide crucial information on time-resolved parental histone dynamics, since they compare only pre- and postreplicated states of chromatin.

By direct visualization of replisome-nucleosome encounters, we demonstrate that, contrary to the prevailing view, replication fork collision with nucleosomes does not always result in an instant parental histone transfer onto daughter strands (Figs. 3 and 4). Three additional outcomes are possible: histone eviction, histone sliding, and replication fork stalling. While histone eviction undoubtedly represents parental nucleosome disassembly, the very first step on the possible histone recycling trajectory, the latter two cases have not been observed before for nucleosome-fork encounter. Histone sliding has two equally probable molecular explanations that, as yet, we cannot distinguish; either a whole nucleosome is pushed ahead of the replication fork or an evicted parental histone is “piggybacking” on the replisome. The piggybacking mechanism is particularly interesting since, if true, it would represent the second intermediate step on the histone transfer pathway, whereby released parental histones are ushered to daughter strands via a series of interactions facilitated by histone chaperones and replisome components, such as FACT, MCM2, Ctf4, or Pol α (*5*, *6*, *9*, *18*–*20*). A third possible explanation for histone sliding is a series of downstream (ahead of the replisome) passive histone transfer steps via the proposed DNA looping mechanism (*6*, *21*, *22*). While we judge this phenomenon less likely in our experimental setup, because of the crowded environment of extracts and increased likelihood of exchange, we cannot discount its contribution to the sliding process. Histone sliding that results in ex situ histone transfer could be of significance for epigenetic memory, which relies on the preservation of positional information (*13*). We note that, although additional dark nucleosomes are present in our experiments (Supplementary Text), the extent of histone sliding on fully chromatinized DNA is likely to be more limited than in the case of lower density λ nucleosomes. At present, it is impossible for us to reliably assess the extent or proportion of histone sliding on high-density nucleosomal templates because of the overlapping histone signals, molecule fluctuations, and other factors. Further studies are needed to identify the underlying molecular basis for the observed sliding behavior.

Replication fork stalling upon collision with a nucleosome has an obvious molecular interpretation—a nucleosome constitutes a roadblock and stops progression of the replisome. Other DNA binding proteins can lead to fork stalling in egg extracts (*31*). Fork stalling is a transient state that, in most cases, terminates in parental histone eviction or recycling. Persistent stalling events (i.e., when replication fork never restarts on the experimental time scale) typically occur when the nucleosome is located at the very end of λ DNA. Because the DNA molecules in our assays are of finite length (48.5 kbp), the likelihood of finding an end-point nucleosome is much higher than for longer DNA, and so the proportion of persistent stalling events in our quantifications must be an overestimate.

### Role of newly synthesized histones in parental histone recycling

To maintain correct nucleosome density on the replicated daughter DNA strands, nucleosomes are assembled from the pool of recycled parental histones and newly synthesized histones. Assuming that all parental histones are reinstated during replication, an equal amount of newly synthesized histones needs to be delivered into the nucleus to restore chromatin structure. This high demand for canonical core histones during S phase is fulfilled through rapid expression of multicopy histone genes, induced at the onset of replication and tightly regulated throughout the cell cycle (*32*). Because histones are highly basic proteins and so have the potential to bind nonspecifically to negatively charged macromolecules, such as DNA and RNA, they are escorted throughout their cellular life by dedicated networks of chaperone proteins (*33*). Histone chaperones ensure their correct folding, control their traffic within the cell (such as nuclear import, nucleosome assembly, and histone degradation), and assist nucleosome dynamics. The deficit or excess of canonical histones was found to inhibit DNA replication and lead to genomic instability in yeast and mammalian cells (*5*, *34*).

*Xenopus* eggs naturally contain high amounts of histones because they need to support the first 12 rounds of DNA replication before the midblastula transition, when transcription is initiated (*28*). Consequently, egg extracts have a significantly higher concentration of “free” histones than an equivalent extract of somatic cells. The quantitative analysis of the replication fork collision with nucleosomes in these extracts revealed that histone eviction is the dominant outcome, approximately three times more likely than parental histone transfer (Fig. 5). Extracts depleted of a large proportion of newly synthesized histones promoted efficient parental histone recycling, increasing its likelihood to ~3:1 over histone eviction. Supplementation of depleted extracts with recombinant histones reversed this effect, resulting in histone eviction prevalence over histone transfer, at ~2:1 likelihood ratio.

**Fig. 6:**
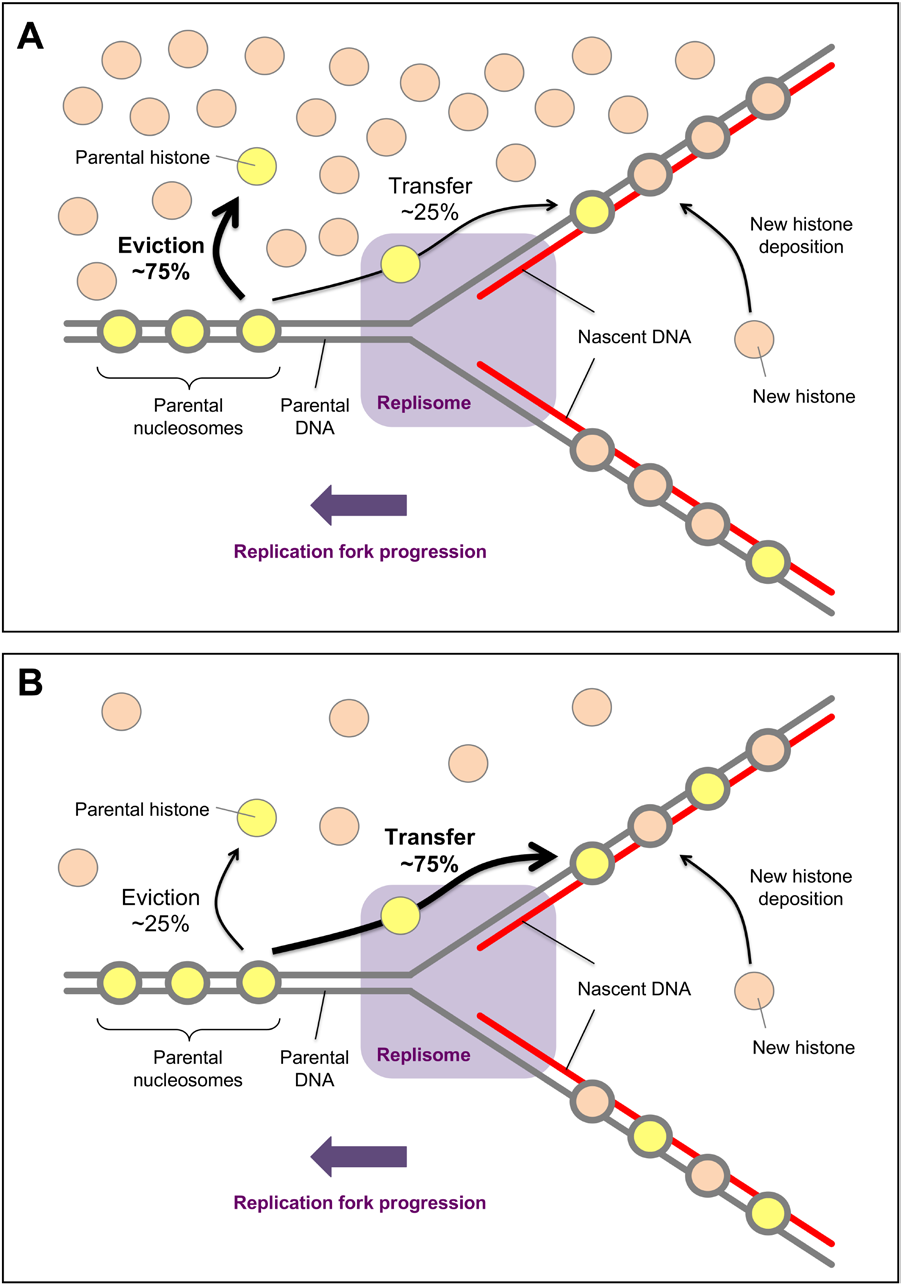
Model of parental histone transfer at high and low concentrations of newly synthesized histones. (**A**) At high concentrations of free histones, upon the encounter with the replication machinery, most of the parental histones are evicted from the DNA and released into the histone pool. (**B**) When the concentration of newly synthesized histones is low, most of the parental histones are recycled at the replication fork. Upon nucleosome disassembly ahead of the replication fork, parental histones are released from the DNA but are kept in the vicinity of the replisome, most likely through a concerted action of histone chaperones, replisome components, and DNA looping. Parental histones are rapidly ushered behind the replication fork where they are deposited onto daughter strands.

Our analysis clearly demonstrates that the efficiency of localized parental histone recycling depends on the concentration of newly synthesized histones. We interpret these results with the following molecular model (Fig. 6). At low concentrations of free histones, most of the parental histones are locally recycled. Upon nucleosome disassembly ahead of the replication fork, parental histones are released from the DNA and remain in the vicinity of the replisome, through a concerted action of histone chaperones and replisome components, which finally deposit parental histones on the daughter DNA. When the concentration of newly synthesized histones is high, most parental histones are released into the milieu and do not get incorporated into replicated DNA. The most probable explanation for such behavior is that the free histones exchange with their parental counterparts en route from parental to nascent DNA, as found for some replisome components (*35*). Although less likely, we cannot rule out the possibility that the pathway of newly synthesized histone deposition takes over in conditions of excessive histone provision and inhibits localized parental histone recycling. Our observations that free histone concentration modulates parental histone recycling during replication are also consistent with the passive histone transfer model, in which histone “handover” is mediated by DNA loop formation. We anticipate that the mechanism of histone recycling in vivo is driven by a coordinated action of various proteins (replisome components, histone chaperones, and chromatin remodelers), which orchestrate intrinsic DNA mechanics at the replication fork.

### Consequences for epigenetic inheritance

Nucleosomes in transcriptionally active and repressed chromatin domains carry specific histone PTMs, which modulate their structure and dynamics (*4*). The key question in the field of epigenetics is whether localized parental histone recycling at the replication fork drives the transgenerational transmission of PTMs and chromatin domain inheritance. Some studies imply that histones carrying either active or repressive marks are accurately recycled during replication, preserving their positional information and allowing PTM transmission to daughter cells (*13*). Others suggest that parental histones in repressed chromatin states are indeed preserved through faithful localized recycling, whereas histones in euchromatin are not (*16*). Therefore, a critical question arises—what molecular mechanism could lead to different patterns of histone inheritance throughout the genome?

One possible explanation is that there are PTM-specific chaperones, which direct parental histones either for local recycling (repressive PTMs) or into the pool of soluble histones (active PTMs). This model, however, seems unlikely given that histones without any PTMs are efficiently transferred to daughter strands during replication supported by purified yeast replisome (in the absence of soluble histones) (*9*). Another possibility, which has been raised previously (*16*), is the difference in replication rate between early-replicating (euchromatin) and late-replicating (heterochromatin) domains (*36*). Our analysis of the replication fork collision with nucleosomes shows that the velocity of the progressing replication fork has no influence on the collision outcome (fig. S8), rendering this explanation less probable. On the basis of our findings that the efficiency of parental histone recycling depends on the concentration of newly synthesized histones (Figs. 5 and 6), we propose an alternative molecular mechanism, in which differential levels of accessible free histones are used to prevent local histone recycling in euchromatin but promote it in heterochromatic regions.

Rapid histone biosynthesis is activated at the beginning of S phase and persists at high levels until the end of S phase, when DNA replication is halted (*32*). However, it remains unknown what the concentration of newly synthesized histones is in the nucleus and how it varies through space and time. Transcriptionally active and silenced chromatin domains display distinct spatial segregation in the nucleus (*37*), and their replication is separated in time (*36*). Recent studies show that associations between heterochromatic regions lead to phase separation of active and repressed chromatin (*38*, *39*). We speculate that the phase boundary could act as a selective barrier to histones and/or the associated chaperones and thus provide distinct regions of histone accessibility within the nucleus during replication. Phase-separated heterochromatin domains would replicate under conditions of limited provision of newly synthesized histones, ensuring efficient localized parental histone transfer at the replication fork, and so its epigenetic inheritance. In the case of transcriptionally active euchromatin, the high local concentration of newly synthesized histones would lead to dispersed redistribution of parental and newly synthesized histones on daughter strands.

## MATERIALS AND METHODS

### Preparation of biotinylated λ DNA

Singly biotinylated λ DNA was prepared as described in (*25*). Doubly biotinylated λ DNA was prepared by mixing 80 μM biotin-14–deoxycytidine triphosphate (Invitrogen, 19524-016), 80 μM biotin-14–deoxyadenosine triphosphate (dATP; Invitrogen, 19518-018), 100 μM deoxythymidine triphosphate (Thermo Fisher Scientific, R0171), 100 μM deoxyguanosine triphosphate (Thermo Fisher Scientific, R0161), λ DNA [130 ng/μl; New England BioLabs, Inc. (NEB), N3011], and Klenow fragment (0.05 U/μl; NEB, M0212S) in provided Klenow buffer. Mixture was incubated at 37°C for 30 min, followed by 15 min at 70°C. λ DNA was purified using QIAquick PCR Purification Kit (Qiagen, 28104) and stored at 4°C. This method introduces multiple biotins at each end of λ DNA (seven biotins at the left end and four biotins at the right end, assuming 100% incorporation of biotinylated deoxynucleoside triphosphates).

### Histone labeling under denaturing conditions

Purified, recombinant *Xenopus* histones were purchased from The Histone Source, Protein Expression and Purification Facility, Colorado State University, and their correct molecular mass was verified by mass spectrometry (Proteomics Science Technology Platform, Francis Crick Institute). Histones H2A-K119C, H2B-T112C, H3-C110A-T80C, and H4-E63C were labeled with either Cy5 maleimide (GE Healthcare, PA25031) or Alexa Fluor 647 C_2_ maleimide (Thermo Fisher Scientific, A20347) using thiol modification of engineered cysteines. Before labeling, histones were reduced and denatured in 20 mM tris-HCl (pH 7.5; Sigma-Aldrich, T1503; and Thermo Fisher Scientific, 10316380), 10 mM tris(2-carboxyethyl)phosphine (TCEP; Sigma-Aldrich, 646547), and 7 M guanidine hydrochloride (Sigma-Aldrich, 50940) for 30 min at room temperature. Each denaturing reaction contained a chosen histone at a concentration of 150 μM in a total volume of 250 μl (equivalent to approximately 0.5 mg of histone). One vial of Cy5 maleimide or 0.5 mg of Alexa Fluor 647 C_2_ maleimide was dissolved in 50 μl of anhydrous dimethyl sulfoxide (DMSO; Invitrogen, D12345) and then mixed dropwise with 250 μl of the denatured histone solution. Labeling reactions were carried out for 2.5 to 3 hours at room temperature and protected from light. β-mercaptoethanol (Sigma-Aldrich, 101458612) was added to a labeling reaction at a 100-fold molar excess of the dye to consume any unreacted species. The quenched reaction was used immediately to refold histone octamer.

### Histone octamer refolding and purification

Histone octamer refolding protocol was adapted from (*40*). Histones were individually reduced and denatured in 20 mM tris-HCl (pH 7.5), 10 mM β-mercaptoethanol, and 7 M guanidine hydrochloride for 3 hours at room temperature. Each denaturing reaction contained a chosen histone at a concentration of 150 μM in a total volume of 250 μl (equivalent to approximately 0.5 mg of histone). Denatured histones H2A, H2B, H3, and H4 were mixed at equimolar ratios and adjusted to a total protein concentration of 1 mg/ml with unfolding buffer [20 mM tris-HCl (pH 7.5), 10 mM β-mercaptoethanol, and 7 M guanidine hydrochloride]. For labeled octamer refolding, a quenched labeling reaction was used instead of a wild-type denatured histone. Denatured histone mix was loaded into a MaxiGeBaFlex dialysis tube (Generon, D045; 8-kDa molecular weight cutoff; 2- to 3-ml capacity) and dialyzed at 4°C against 2 liters of 10 mM tris-HCl (pH 7.5), 1 mM ethylenediaminetetraacetic acid disodium salt dihydrate (EDTA; Sigma-Aldrich, E5134), 5 mM β-mercaptoethanol, and 2 M NaCl (Sigma-Aldrich, S9888). Refolding buffer was changed at least three times for unlabeled octamer and four times for fluorescently labeled octamers (first, overnight; second, 8 hours; third, overnight; and fourth, 8 hours).

Refolded histone mixture was recovered from the dialysis device and concentrated to approximately 0.3 ml using a VivaSpin 500 centrifugal concentrator [Sartorius, VS0121; 30-kDa molecular weight cutoff; polyethersulfone (PES)] at 2°C, 15,000*g*. Concentrated sample was resolved on a Superdex 200 Increase GL10/300 column (GE Healthcare, 28-9909-44), over 1.1 column volume of refolding buffer [10 mM tris-HCl (pH 7.5), 1 mM EDTA, 5 mM β-mercaptoethanol, and 2 M NaCl] at 0.3 ml/min flow rate, 4°C. Fractions containing stoichiometric octamer, as verified by SDS–polyacrylamide gel electrophoresis, were pooled and concentrated using the VivaSpin 500 centrifugal concentrator (30-kDa molecular weight cutoff; PES). Octamer concentration and labeling efficiency were estimated spectrophotometrically from the absorbance measurement at 276 and 650 nm. Octamer was flash-frozen in liquid nitrogen and stored at −80°C.

### Histone octamer labeling under native conditions

Histone octamer containing H3-K36C^Cy5^ was prepared by thiol modification under native conditions. Octamer containing unlabeled H3-K36C was refolded and purified as described above, but the unfolding and refolding buffers contained TCEP, instead of β-mercaptoethanol, as a reducing agent. Octamer (0.5 mg) was adjusted to a concentration of 1 mg/ml with refolding buffer. One vial of Cy5 maleimide was dissolved in 50 μl of anhydrous DMSO and then mixed dropwise with the octamer solution. Labeling reactions were carried out overnight at 2°C, protected from light. β-mercaptoethanol (Sigma-Aldrich, 101458612) was added to a labeling reaction at a 100-fold molar excess of the dye to quench any unreacted species. Excess dye was removed using Micro Bio-Spin P-30 Columns (Bio-Rad, 7326202), pre-equilibrated with refolding buffer. Octamer concentration and labeling efficiency were estimated spectrophotometrically from the absorbance measurement at 276 and 650 nm. Octamer was flash-frozen in liquid nitrogen and stored at −80°C.

### Nucleosome reconstitution

Nucleosome reconstitution was performed by a NaCl gradient dialysis method. For each reconstitution reaction, 1 μg of DNA was mixed with a desired molar excess of histone octamer (from 0 to 300 for λ DNA) in 10 mM tris-HCl (pH 7.5), 1 mM EDTA, and 2 M NaCl, to a final volume of 100 μl, and incubated on ice for 30 min. Samples were then transferred into Slide-A-Lyzer MINI dialysis units (Thermo Fisher Scientific, 96570) and dialyzed overnight against 1 liter of 10 mM tris-HCl (pH 7.5), 1 mM EDTA, and 1 M NaCl. Second dialysis was performed for 8 hours against 1 liter of 10 mM tris-HCl (pH 7.5), 1 mM EDTA, and 0.75 M NaCl, before the final overnight dialysis against 1 liter of 10 mM tris-HCl (pH 7.5), 1 mM EDTA, and 20 mM NaCl. Reconstituted nucleosomes were recovered from the dialysis devices and stored at 4°C. Samples containing fluorescently labeled histones were protected from light at each step.

### Electrophoretic mobility shift assay

Naked λ DNA or λ nucleosomes (100 ng) in 10 mM tris-HCl (pH 7.5), 1 mM EDTA, 20 mM NaCl, and 10% glycerol (Thermo Fisher Scientific, BP229-1) were resolved on a 0.5% agarose (Denville Scientific Inc., CA3510-8) gel in 20 mM tris and 20 mM boric acid (Fisher Chemical, B/3800/53) for 120 min at 100 V. After electrophoresis, DNA was stained with SYBR Gold nucleic acid gel stain (Thermo Fisher Scientific, S11494) following the manufacturer’s protocol. Gels were imaged using a fluorescent image analyzer, FLA-5000 (Fujifilm). Samples containing fluorescently labeled histones were protected from light at each step.

### Native MNase protection assay

Naked λ or λ nucleosomes (100 ng) in 10 mM tris-HCl (pH 7.5), 1 mM EDTA, and 20 mM NaCl were supplemented with MNase buffer (NEB, M0247S), following the manufacturer’s instructions, and then digested with 10 gel units of MNase (NEB, M0247S) for 10 min at room temperature. Digest was quenched by adding EDTA to a concentration of 25 mM, and 10% glycerol was used as a loading agent. Digested samples were resolved on a 1.5% agarose gel in 20 mM tris and 20 mM boric acid for 120 min at 100 V. After electrophoresis, DNA was stained with SYBR Gold nucleic acid gel stain and imaged using the fluorescent image analyzer FLA-5000. Samples containing fluorescently labeled histones were protected from light at each step.

### Denaturing MNase protection assay

In the denaturing MNase protection assay, samples were prepared, digested, and quenched as described for native assay. Upon quenching with EDTA, each sample was supplemented with SDS (Sigma-Aldrich, 436143) to a concentration of 0.8% and 0.8 U of proteinase K (NEB, P8107S). Protein digest was conducted at 37°C for 1 hour. Samples were supplemented with glycerol to 10% and resolved on a 1.5% agarose gel in 100 mM tris, 100 mM boric acid, and 2 mM EDTA (TBE). DNA was stained with SYBR Gold nucleic acid gel stain and imaged using the fluorescent image analyzer FLA-5000.

### *X. laevis* egg extracts preparation

HSS and NPE were prepared as described previously (*24*) and stored at −80°C. Before both bulk- and single-molecule replication assays, each 33-μl aliquot of HSS was supplemented with 250 ng of nocodazole (Sigma-Aldrich, M1404) and 1 μl of an adenosine triphosphate (ATP) regeneration system, containing 650 mM phosphocreatine (Sigma-Aldrich, P7936), 65 mM ATP (pH 7.0; Sigma-Aldrich, A2754) and creatine phosphokinase (0.161 mg/ml; Sigma-Aldrich, C3755). Similarly, each 16-μl aliquot of NPE was supplemented with 0.5 μl of ATP mix. Activated extracts were centrifuged for 5 min at 16,000*g*, room temperature and used in replication assays. All *Xenopus* work fully complied with the UK Animals (Scientific Procedures) Act 1986 as implemented by the Francis Crick Institute.

### Histone depletion from *Xenopus* egg extracts

Fifty microliters (bed volume) of protein A Sepharose (PAS; GE Healthcare, GE17-1279-01), prewashed with ice-cold phosphate-buffered saline (PBS; Gibco, 70011044; six times with 300 μl), was mixed with 300 μl of anti–H4-K12Ac antibody solution (1.6 mg/ml) in PBS and then incubated overnight at 4°C, 20 rpm. PAS loaded with an antibody was washed four times with 300 μl of cold PBS and three times with 300 μl of cold egg lysis buffer (ELB) by centrifugation. HSS-NPE mix (200 μl; extracts were not supplemented with ATP mix, but nocodazole was added into HSS to prevent microtubule polymerization) at 1:1 volume ratio was next mixed with 50 μl (bed volume) of antibody-loaded PAS and incubated for 1 hour at 4°C, 20 rpm. Extracts were separated from PAS by spinning through a nitex column, as described in (*24*). Cleared extracts were mixed with 34 μl (bed volume) of PAS, prewashed with cold PBS (six times with 300 μl) and ELB (three times with 300 μl), and incubated for 45 min at 4°C, 20 rpm. This step ensures that any leftover antibody is captured and removed from extracts. Last, depleted extracts were clarified on a nitex spin column and either used immediately in replication assays or snap-frozen in liquid nitrogen and stored at −80°C.

### Bulk replication assay

Naked pBRII (pBlueScript II; Agilent Technologies, 212205) plasmid and pBRII containing fluorescent nucleosomes (at a saturation level equivalent with λ nucleosomes in single-molecule replication assays) labeled at H3-K36C^Cy5^ or H4-E63CA^647^ were adjusted to a DNA concentration of 18 ng/μl with ELB [2.5 mM MgCl_2_, 50 mM KCl, and 10 mM Hepes-KOH (pH 7.7); Sigma-Aldrich, M8266; Sigma-Aldrich, P9333; Sigma-Aldrich, H3375], supplemented with ATP mix (1 μl per 16 μl of DNA in ELB), and then mixed at 1:1 volume ratio with activated HSS. Equivalent reactions were set up with HSS supplemented and preincubated (5 min at room temperature) with 4 μM geminin, as replication-negative controls. All samples were incubated for 15 min at room temperature to promote origin licensing. Activated NPE (16 μl) was supplemented with 0.2 μl of 10 mCi/ml [α-^32^P]dATP (3 kCi/mmol; PerkinElmer, BLU512H250UC). [α-^32^P]dATP gets incorporated into nascent DNA strands during replication and thus allows to track the progress of replication in time. At 8, 15, and 30 min after NPE was introduced, a 2.5-μl aliquot of a replication reaction was stopped by mixing in 5.0 μl of solution containing 25 mM tris-HCl (pH 8.0), 2% SDS, 75 mM EDTA, and proteinase K (8 U/ml), and incubated at 37°C for 1 hour. Replication reactions were separated on a 0.8% agarose gel in TBE at 90 V, room temperature. Gel was dried and visualized using the fluorescent image analyzer FLA-5000 in a phosphorescence mode.

### Expression and purification of Fen1-KikGR

Fen1-KikGR was expressed and purified from *E. coli* as described in (*23*).

### Single-molecule replication assay

Microfluidic flow cells with PEGylated and streptavidin-functionalized glass surface were prepared as described previously (*25*). Flow cells were mounted on a Nikon Eclipse Ti motorized inverted microscope, equipped with a 100× high–numerical aperture TIRF objective (SR Apo TIRF 100× 1.49 Oil; Nikon) and the Perfect Focus System and supported by an LU-N4 laser unit (Nikon), providing four lasers: 405, 488, 561, and 640 nm (15-mW output power at the fiber end). Images were recorded using a 512 × 512 pixel, back-illuminated, electron-multiplying charge-coupled device camera (iXon DU-987, Andor Technology; 3-MHz pixel readout rate, 14-bit digitization, and 300× electron multiplier gain) controlled by NIS-Elements software (Nikon). The pixel size was 160 × 160 nm. All buffers and solutions were thoroughly degassed immediately before use. Flow was controlled by an automated syringe pump (Pump 11 Elite; Harvard Apparatus, 70-4505). All experiments were conducted at room temperature.

Before DNA immobilization, microfluidic channels were washed with blocking buffer containing 20 mM tris (pH 7.5), 50 mM NaCl, 2 mM EDTA, and BSA (albumin from bovine serum; 0.2 mg/ml; Sigma-Aldrich, A7906). For immobilization of singly biotinylated λ nucleosomes, 125 μl of DNA or nucleosome solution at a concentration of 0.1 ng/μl in blocking buffer was passed through the channel at a flow rate of 25 μl/min. DNA was incubated in the channel for 10 min, and any unbound molecules were removed by washing with 250 μl of blocking buffer at 50 μl/min flow rate. Doubly biotinylated naked λ or λ nucleosomes were immobilized by passing through 500 μl of DNA or nucleosome solution at a concentration of 0.1 ng/μl in blocking buffer at a flow rate of 100 μl/min. This procedure immobilizes λ DNA and λ nucleosomes to approximately 70% of their respective, maximally stretched contour lengths. Cy5- or Alexa Fluor 647–labeled histones within immobilized nucleosomes were imaged using a 640-nm laser at 10% power, 100-ms exposure time, and ZT405/488/561/647rpc dichroic (Chroma). Tethered DNA molecules were stained with 5 nM SYTOX Orange (Thermo Fisher Scientific, S11368) in blocking buffer and imaged using a 560-nm laser at 5% power, 100-ms exposure time, and ZT405/488/561/647rpc dichroic. To remove SYTOX Orange, a flow cell was washed extensively with blocking buffer, 0.5 to 1.0 ml at a flow rate of 50 μl/min. Immediately before licensing, ELB supplemented with casein (Sigma-Aldrich, C4765) and BSA to a final concentration of 1 mg/ml was introduced into the channel at 25 μl/min for 3 min.

For licensing of the immobilized DNA, an aliquot of activated and spun-down HSS (see *X. laevis* egg extract preparation) was transferred to a fresh tube, supplemented with a short linear “carrier” DNA (preannealed oligos 5′-GCA GCA ACA GAA GCC ATG GAT GCC CTG AC-3′ and 5′-GTC AGG GCA TCC ATG GCT TCT GTT GCT GC-3′) to a concentration of 10 ng/ul and incubated for 5 min. HSS was introduced into the channel at a flow rate of 10 ul/min over 2.5 min and incubated for a further 12.5 min.

As the extract reached immobilized λ nucleosomes in the flow cell, thermal fluctuations of individual molecules became gradually reduced because of the DNA being bound by extract proteins, including native histones. HSS is known to efficiently chromatinize naked DNA. Singly tethered λ molecules containing a few fluorescent nucleosomes became fully chromatinized in HSS (because of deposition of native “dark” histones) and compacted the individual molecules to a diffraction-limited spot, which do not stretch under buffer flow. Naked λ DNA (48.5 kbp) accommodates approximately 240 nucleosomes, assuming 0.2 kbp per nucleosome. In the case of doubly tethered, stretched λ nucleosomes, the chromatinization is limited by the slack within the molecule. On the basis of the measured average contour length for λ nucleosomes, stretched to ~70% of their maximally stretched form, we estimate that approximately 70 nucleosomes (both fluorescent and dark) were present on doubly tethered λ nucleosomes in our assays. During licensing in HSS, Cy5- or Alexa Fluor 647–labeled histones were imaged using a 640-nm laser at 5% power, 100-ms exposure time, and ZT405/488/561/647rpc dichroic (Chroma). Images were collected for 25 different fields of view (5 × 5 grid; 512 × 512 pixels per field of view) at an 11-s interval between frames.

While the licensing reaction was taking place, replication extracts were prepared by mixing activated HSS, NPE, and ELB at 1:1:1 volume ratio. The replication mix was further supplemented with the pBRII plasmid to a final concentration of 5 ng/μl, Fen1-KikGR to 2.5 μM, and an oxygen-scavenging system [i.e., glucose to 40 mM, pyranose oxidase to 2.5 U/ml, and catalase to 120 U/ml; Sigma-Aldrich, G8270; Sigma-Aldrich, P4234-250UN; Sigma-Aldrich, C30-100MG; (*41*)]. For unrestricted origin firing (replication from multiple origins), 40 μl of this mix was drawn into the channel at 10 μl/min flow rate. To achieve replication from single origins, the mix was split into two 20-μl aliquots. One aliquot was immediately drawn into the channel at 10 μl/min for 2 min to initiate replication of licensed and immobilized DNA molecules. The other aliquot was supplemented with p27^Kip^, a Cdk2 inhibitor, to a concentration of 0.1 μg/μl and introduced into the channel when about one or two origins per template fired, typically between 4 and 8 min from the moment the first extract was drawn in. During replication, Cy5- or Alexa Fluor 647–labeled histones were imaged using a 640-nm laser at 5% power, 100-ms exposure time, and ZT405/488/561/647rpc dichroic. Fen1-KikGR–decorated replication bubbles were imaged using a 488-nm laser at 5% power, 100-ms exposure time, and ZT405/488/561/647rpc dichroic. Unless stated otherwise, images were collected for 36 different fields of view (6 × 6 grid; 512 × 512 pixels per field of view) at a 1-min interval between frames.

For replication in extracts depleted of endogenous histones H4 and H3, DNA template immobilization and licensing were conducted as described above. H4/H3-depleted HSS-NPE mix (16 μl) was supplemented with 0.5 μl of ATP mix and centrifuged for 5 min at 16,000*g*, room temperature. The activated extract mix was next transferred to a fresh tube and supplemented with pBRII to a final concentration of 5 ng/μl, Fen1-KikGR to 2.5 μM, and an oxygen-scavenging system (i.e., glucose to 40 mM, pyranose oxidase to 3 U/ml, and catalase to 90 U/ml). In the case of replication experiments in extracts depleted of endogenous histones but supplemented with recombinant histones, the activated mix was additionally supplemented with histones H3 and H4 to a final concentration of 20 μM. The volume was adjusted to 20 μl with ELB, the mixture was drawn into the channel, and imaging was conducted as described for undepleted extracts. Depleted extracts showed lower overall origin firing efficiency, relative to undepleted extracts, and so did not require p27^Kip^ supplementation for individual bubble growth tracking during replication.

For replication in the absence of Fen1-KikGR, DNA template immobilization and licensing were conducted as described above. Replication extracts were prepared by mixing activated HSS, NPE, and ELB at 1:1:1 volume ratio. The replication mix was further supplemented with pBRII to a final concentration of 5 ng/μl, dig-dUTP (Roche, 11093088910) to 1.7 mM, and an oxygen-scavenging system (i.e., glucose to 40 mM, pyranose oxidase to 3 U/ml, and catalase to 90 U/ml). The mix was split into two 20-μl aliquots. One aliquot was immediately drawn into the channel at 10 μl/min for 2 min to initiate replication. The other aliquot was supplemented with p27^Kip^ to a concentration of 0.1 μg/μl and introduced into the channel at 9 min from the moment the first extract was drawn in. Replication elongation was allowed to proceed for next 31 min before a buffer containing 20 mM tris (pH 7.5), 10 mM EDTA, and 0.5 M NaCl was flown in at a rate of 20 μl/min over 10 min to wash out the extracts. The flow cell was next washed with 250 μl of blocking buffer at 50 μl/min flow rate. Three hundred fifty microliters of a 0.2 ng/μl solution of fluorescein-labeled anti-digoxigenin Fab fragments from sheep (anti-dig Ab^Fluor^; Roche, 11207741910) in blocking buffer, supplemented with casein (1 mg/ml) and BSA (1 mg/ml), was introduced into the chamber at a flow rate of 10 μl/min. The flow cell was next washed with 100 μl of blocking buffer at 20 μl/min flow rate. Last, λ DNA was stained with a 5 nM SYTOX Orange solution in blocking buffer, drawn into the cell at a rate of 20 μl/min. Cy5-labeled histones were imaged using a 640-nm laser at 5% power, 100-ms exposure time, and an ET700/50m emission filter (Chroma). Nascent DNA, decorated with anti-dig Ab^Fluor^, was imaged using a 488-nm laser at 2% power, 100-ms exposure time, and an ET525/50m emission filter (Chroma). SYTOX was excited using a 561-nm laser at 5% power, 100-ms exposure time, and an ET600/50m emission filter (Chroma).

### Single-molecule data processing, analysis, and quantification

All data were recorded in a 5 × 5 or 6 × 6 field-of-view grid format. Data were first denoised using “advanced denoising” in NIS-Analysis (Nikon), with a denoising power set to 0 for all channels. Background was corrected using a rolling ball algorithm (NIS-Analysis, Nikon), with a ball radius set to 0.96 μm. Grid images were next split to individual fields of view, which were subsequently corrected for drift using “align” in NIS-Analysis. Regions of interest were selected by hand, cropped, and, if needed, rotated using Fiji. Kymograms were generated using “montage” in Fiji.

For intensity analysis during licensing in HSS, the intensity plots were generated in Fiji for individual molecules between 3 and 14 min of incubation time. Data were normalized to background (“0”) and maximum intensity value (“1”). Average intensity profiles were generated for each tested nucleosomal template, with a mean fluorescence value and SD calculated at each time point. The mean value traces were then fitted to a single exponential decay model using Prism (GraphPad).

Replication fork velocities were calculated by measuring the distance traveled by an individual fork over time, in micrometers per minute. Velocities were next converted to nucleotides per minute based on the measured average length of λ DNA from SYTOX staining. Mean fork velocities and associated SDs were calculated from a Gaussian fit to a histogram (GraphPad Prism).

For the analysis of fork-nucleosome collision outcomes, a number of criteria were implemented to ensure their reliable assignment and quantification. Only well-separated stretched λ molecules were included in the analysis. Events occurring away from the replication forks (i.e., replication-independent histone losses) were not counted in the statistics. Histones that displayed thermal fluctuations inconsistent with the stretched λ DNA molecule were also excluded from the analysis. For example, histones adsorbed to the surface display a very rigid trail in kymograms, in contrast to free DNA-bound histones, which undergo slight fluctuations throughout data collection. Similarly, if a broken singly tethered λ DNA is located close to a doubly tethered λ DNA, then its nucleosomes may, at a first glance, appear as part of the doubly tethered molecule but are usually distinguishable through local fluctuations over time. Histone eviction was defined by the loss of histone fluorescence in the next time frame upon fork encounter. Histone transfer was assigned when, upon fork encounter, histone-associated fluorescence was incorporated into the replication bubble and could be followed for at least three subsequent time frames (3 min). Histone sliding was determined by a unified histone-fork movement over at least three pixels (0.48 μM, ~2.3 kbp). Replication fork stalling was assigned if a fork movement was arrested by a static (within one pixel) histone fluorescence for at least three time frames (3 min). Stalling events on nucleosomes showing particularly high histone fluorescence (over three times higher than the local average) were excluded from the analysis as they are likely to represent multiple nucleosomes on singly tethered DNA or higher-order local structure on doubly tethered DNA molecules. For the overall outcome quantification, all assigned events were counted, including the secondary events; for example, if a histone sliding was followed by eviction, then both sliding and eviction would be included in the quantification. A separate secondary outcome quantification was also conducted to gain insight into the outcome probability of histone sliding and replication fork stalling.

## Supplementary Material

abc0330_Movie_S13.avi

abc0330_Movie_S5.avi

abc0330_Movie_S15.avi

abc0330_Movie_S8.avi

abc0330_Movie_S6.avi

abc0330_Movie_S12.avi

abc0330_Movie_S14.avi

abc0330_Movie_S3.avi

abc0330_Movie_S17.avi

abc0330_Movie_S4.avi

abc0330_Movie_S16.avi

abc0330_SM.pdf

abc0330_Movie_S1.avi

abc0330_Movie_S9.avi

abc0330_Movie_S7.avi

abc0330_Movie_S11.avi

abc0330_Movie_S10.avi

abc0330_Movie_S2.avi

## SUPPLEMENTARY MATERIALS

Supplementary material for this article is available at http://advances.sciencemag.org/cgi/content/full/6/38/eabc0330/DC1
