# Supplementary material for: Single-molecule imaging reveals control of parental histone recycling by free histones during DNA replication
Source: Sci Adv. 2020 Sep 18;6(38):eabc0330. doi: 10.1126/sciadv.abc0330 (PMC7500940; doi:10.1126/sciadv.abc0330)
Supplement: abc0330_SM.pdf [file abc0330_SM.pdf]

Science Advances
AAAS

advances.sciencemag.org/cgi/content/full/6/38/eabc0330/DC1

Supplementary Materials for

Single-molecule imaging reveals control of parental histone recycling by free histones during DNA replication

D. T. Gruszka, S. Xie, H. Kimura, H. Yardimci*

*Corresponding author. Email: hasan.yardimci@crick.ac.uk

Published 18 September 2020, *Sci. Adv.* **6**, eabc0330 (2020)
DOI: 10.1126/sciadv.abc0330

The PDF file includes:

Supplementary Text
Figs. S1 to S8
Tables S1 and S2
Legends for movies S1 to S17

Other Supplementary Material for this manuscript includes the following:

(available at advances.sciencemag.org/cgi/content/full/6/38/eabc0330/DC1)

Movies S1 to S17

Supplementary Text

Extract-induced chromatinization of fluorescent λ nucleosomes in single-molecule replication assays

Xenopus egg extracts have a well-characterized ability to chromatinize naked DNA (Banaszynski *et al* Methods 2010; DOI: 10.1016/j.ymeth.2009.12.014). In our single-molecule replication assays, upon introduction of HSS, additional dark nucleosomes are assembled on fluorescent λ nucleosomes from endogenous histones, present in extracts at micromolar concentrations (fig. S3A). The extent of extract-induced chromatinization is different for singly- and doubly-tethered molecules in a microfluidic chamber.

In the case of singly-tethered fluorescent λ nucleosomes, the only limit to chromatinization is the amount of naked DNA available. Upon introduction of HSS, all naked DNA regions are prone to being occupied with dark nucleosomes and other DNA binding proteins present in extracts. In our assays, extract-induced chromatinization of singly-tethered fluorescent λ nucleosomes leads to a complete compaction of individual molecules (to a diffraction-limited fluorescent spot), typically within 10-15 sec from the moment extract reached the chamber. Singly-tethered molecules compacted in extracts do not stretch under the flow of native buffers. Naked λ DNA (48.5 kbp) can accommodate approximately 240 nucleosomes in total, assuming 0.2 kbp per nucleosome.

In the case of doubly-tethered, stretched λ nucleosomes, extract-induced chromatinization is additionally limited by the slack within the molecule (only the 'loose' DNA can be chromatinized). Based on the measured average contour length for naked λ and low-density λ nucleosomes, we estimate that ~30 % of λ DNA is available for nucleosome assembly. This suggests that ~70 nucleosomes (both fluorescent and dark; assuming 0.2 kbp per nucleosome) can be present on low-density fluorescent nucleosome templates in our assays (used in experiments presented in Fig. 3-5). As a point of reference, we typically see between 5 and 15 fluorescent nucleosomes per DNA molecule, which leaves room for about 55-65 dark nucleosomes. Please note that these calculations do not take into account DNA compaction caused by binding of other proteins from extracts. Indeed, upon introduction of HSS, dark histone loading inevitably competes with other binding events, including pre-replication complexes assembly. The actual number of dark nucleosomes may therefore deviate from the above estimate.

Loss of fluorescent histones during licensing and replication in extracts

Fluorescent λ nucleosomes are stable in buffer. During licensing in HSS, histone-associated fluorescence is lost at a significantly faster rate (Fig. 2) than the measured rate of photobleaching in buffer (fig. S2). We attribute this loss to the exchange of fluorescent histones with their native, unlabelled counterparts, present in HSS at ~1-6 μM concentration (fig. S3A). This process is likely mediated by histone chaperones and chromatin remodelers (Onikubo and Shechter Int J Dev Biol 2016; DOI: 10.1387/ijdb.130188ds). Histones H2A and H2B exchange approximately three-times faster than histones H3 and H4 (Fig. 2). For low density nucleosome λ templates (containing ~5-15 fluorescent nucleosomes upon immobilization in buffer), about a half of fluorescent histones H3 and H4 are lost, on average, during the 15-minute licensing reaction (Fig. 2).

Upon introduction of NPE, further loss of histone fluorescence is observed due to replication-independent (histone loss away from the replication fork) and replication-induced (histone eviction at the replication fork) processes. The two processes contribute more or less equally to the overall loss of histone fluorescence during replication (fig. S4F) but nevertheless can be readily distinguished in real-time replication data for doubly-tethered λ nucleosomes (for example, see Fig. 4E). Only replication fork-associated histone eviction events are counted in the collision outcome statistics presented in Fig. 5. It is possible that some of these evictions might not be caused by the replisome arrival but other replication-independent processes (e.g. photobleaching or exchange of fluorescent histones with their native counterparts from extracts). However, we estimate the likelihood of such a coincidence to be low, and not significant enough to affect the overall statistical analysis of collision outcomes. For example, in regular extracts, we counted 107 and 54 histone eviction events at the replication forks for nucleosomes containing H4-E63C^{A647} and H3-K36C^{Cy5} (Fig. 5), respectively, while the number of replication-independent losses was 128 and 63, respectively (for the same sets of data). Given that replication-dependent histone eviction and replication-independent histone loss occur at equal probabilities (fig. S4F), this comparison

suggests that most of the replication-independent losses are captured in our real-time data (but are excluded from the statistical analysis of fork-nucleosome collision outcome), and so their coincidental action at the replication fork is likely to be negligible. Regarding the effect of photobleaching specifically at the replication fork, we can estimate its contribution through the following analysis. In regular extracts, we observe 107 histone evictions out of 266 collision outcomes for nucleosomes containing H4-E63C^{A647} (40.2% eviction frequency; Fig. 5). As discussed above, apart from replication fork-associated evictions, we also observe about an equal number of replication-independent histone losses (fig. S4F). The overall rate of photobleaching over the 60-minute replication reaction is estimated as ~13 % loss of total fluorescence (fig. S2) and is likely to affect both replication-dependent and replication-independent histone losses equally. Therefore, assuming the worst-case scenario, in which 6.5 % of all observed fork-associated evictions were due to photobleaching (in other words photobleaching was always coincidental with the arrival of the replication fork), 7 out 107 evictions would be false positives. This would reduce the frequency of eviction from 40.2 % to 37.6 % and would not affect the interpretation of our data. We anticipate that the actual number of false positives due to photobleaching is likely to be lower than this worst-case scenario.

Supplementary Figures

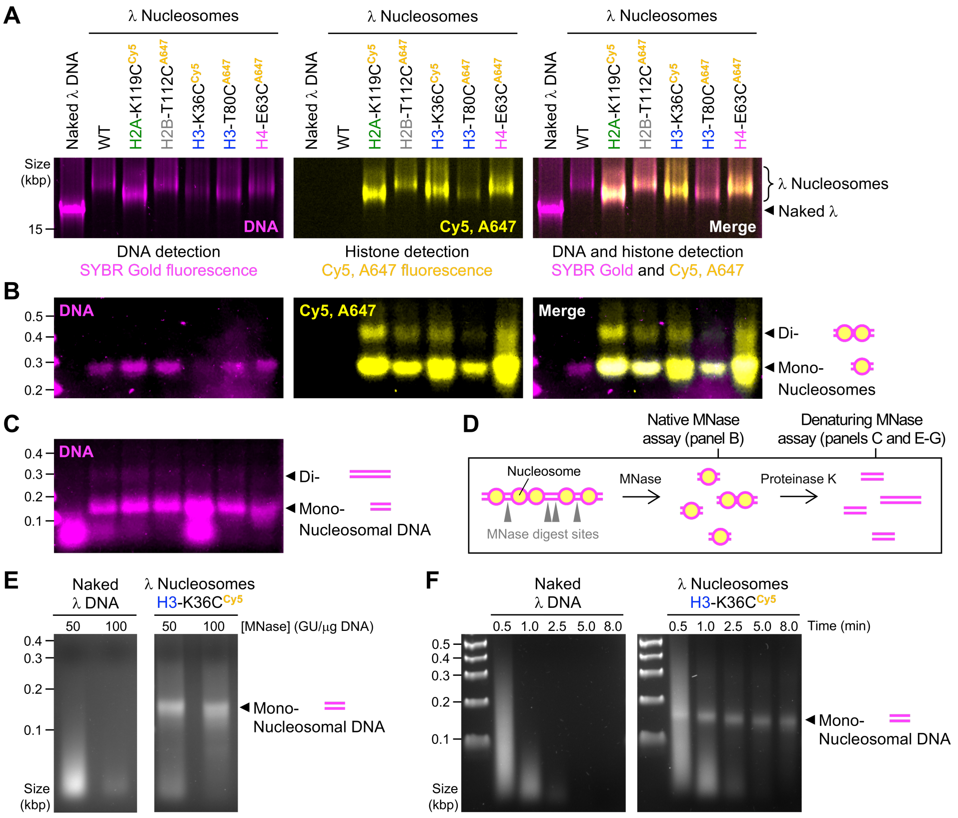

Fig. S1. Assembly of fluorescent nucleosomes on λ DNA. (**A**) Electrophoretic mobility shift assay (EMSA) for WT and fluorescently-labelled nucleosomes reconstituted on λ DNA at ~1:150 DNA:octamer ratio. Left panel shows SYBR Gold staining of the DNA (magenta), central panel shows Cy5 and A647 fluorescence signal (yellow) of labelled histones and right panel is the composite of both detection modes. Naked λ DNA (~48.5 kbp, first lane) migrates through 0.5 % agarose faster than nucleosomal λ templates, containing either WT or fluorescently-labelled histones. (**B**) Native micrococcal nuclease (MNase) protection assay for WT and fluorescently-labelled nucleosomes reconstituted on λ DNA at ~1:150 DNA:octamer ratio. MNase preferentially digests unprotected DNA in linker regions between nucleosomes (see also panel D). Products of MNase digest were resolved in 1.5 % agarose under native conditions revealing intact mono- and di-nucleosomes for nucleosomal templates and complete digest of naked λ DNA (first lane). Signal detection as in panel A. (**C**) Denaturing MNase protection assay for WT and fluorescently-labelled nucleosomes reconstituted on λ DNA at ~1:150 DNA:octamer ratio. Here, products of MNase digest were first deproteinated with proteinase K (see also panel D) in the presence of SDS and then resolved in 1.5 % agarose, yielding DNA fragments protected by mono- (~150 bp band) and di-nucleosomes (~300 bp band) for nucleosomal templates, and short (<100 bp) fragments for naked λ DNA (first lane). (**D**) Schematic overview of native and denaturing MNase protection assays. (**E**) Denaturing MNase protection assay for naked λ DNA and λ nucleosomes containing H3-K36C^{Cy5} (reconstituted at 1:50 DNA:octamer ratio), performed at two different concentrations of the MNase enzyme. Digest products were deproteinated, resolved on a 2.5 % agarose and visualized using GelRed. (**F**) MNase digestion time course for naked λ DNA and λ nucleosomes containing H3-K36C^{Cy5} (reconstituted at 1:50 DNA:octamer ratio). Digest products were deproteinated, resolved on a 2.5 % agarose and visualized using GelRed.

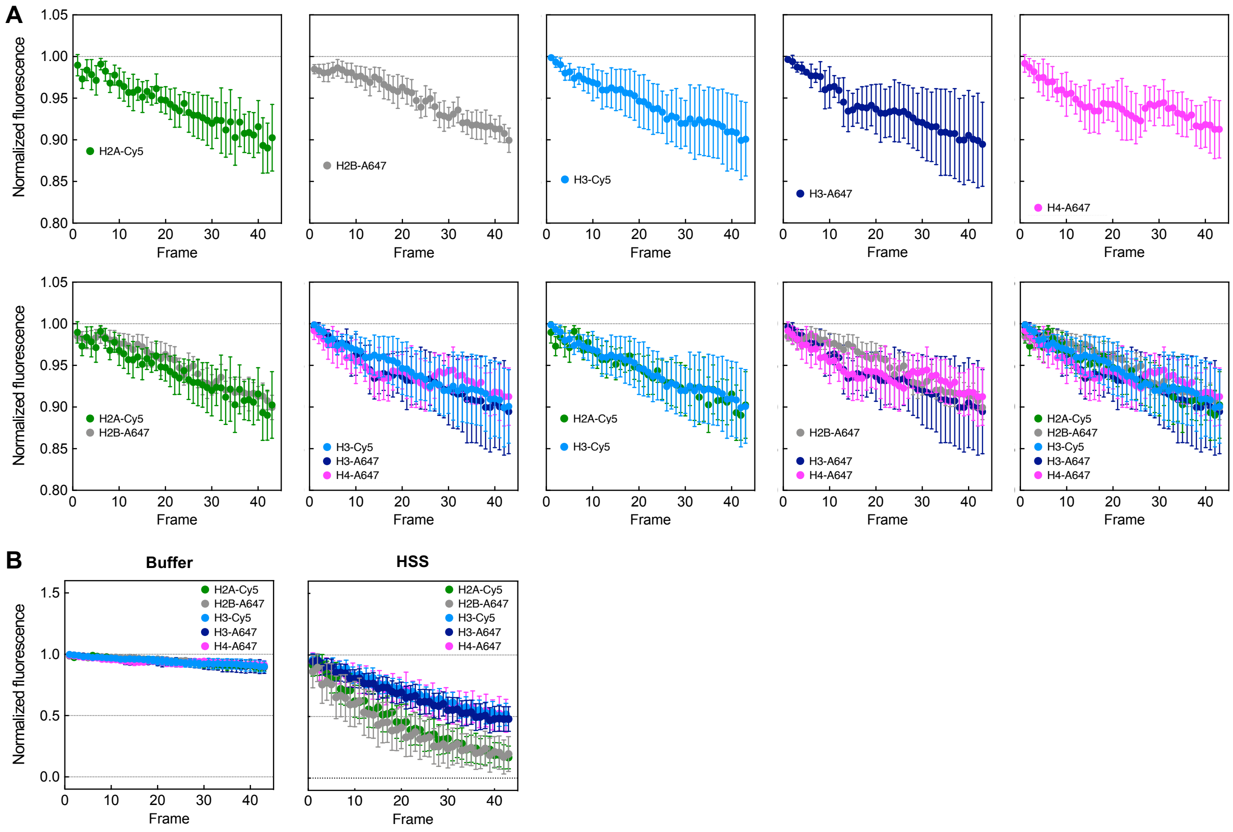

Fig. S2. Photobleaching of fluorescent λ nucleosomes in buffer. (**A**) Plots showing the mean loss of fluorescent signal for λ nucleosomes (H2A-K119^{Cy5}, H2B-T112C^{A647}, H3-K36C^{Cy5}, H3-T80C^{A647} and H4-E63C^{A647}; all labelled and color-coded) during imaging in buffer. Imaging parameters were equivalent to those used during imaging in HSS, presented in Fig. 2 (640 nm laser at 5% power, 100 ms exposure time, 43 frames). 10 fields of view were analyzed for each data set. Decay traces for individual fields of view were normalized to background ('0') and maximum values of fluorescence ('1'). A mean fluorescence value and standard deviation were calculated and plotted for each frame. (**B**) Same-scale comparison of the mean loss of fluorescence signal for λ nucleosomes during imaging in buffer and HSS. The loss of histone fluorescence signal observed in HSS does not result from photobleaching.

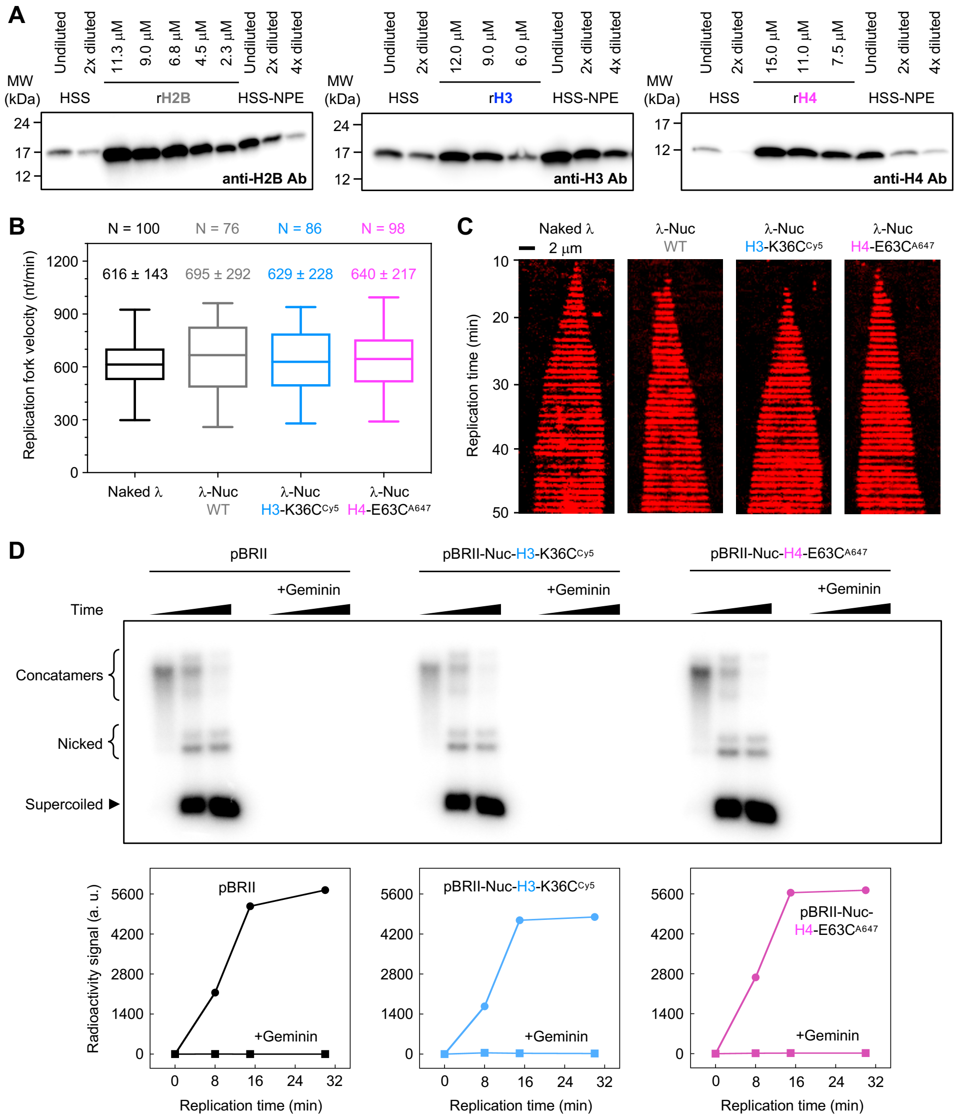

Fig. S3. Replication of λ nucleosomal templates in *Xenopus* egg extracts. (**A**) Western blots used to estimate the concentration of histone H2B, H3 and H4 in *Xenopus* egg extracts. (**B**) Box-and-whisker Tukey plot of replication fork velocities for naked λ and λ nucleosomes containing WT octamer, octamer labelled at H3-K36C^{Cy5} and octamer labelled at H4-E63C^{A647}. Velocities were calculated from real-time single-molecule experiments. Values above the box plots indicate the mean replication fork velocity extracted from the Gaussian fit, plus and minus standard deviation. The number of values analyzed per data set (N) is also shown. (**C**) Kymograms of Fen1-KikGR fluorescence indicating growth of replication bubble over time for naked λ and λ nucleosomes containing WT octamer, octamer labelled at H3-K36C^{Cy5} and octamer labelled at H4-E63C^{A647}. (**D**) Bulk replication assay for naked pBRII plasmid and pBRII plasmid containing nucleosomes labelled at H3-K36C^{Cy5} or H4-E63C^{A647}. For each replicated template, a negative control is also presented (+Geminin). Quantification of the replication efficiency, as measured by the radioactivity signal, is presented in the lower panel.

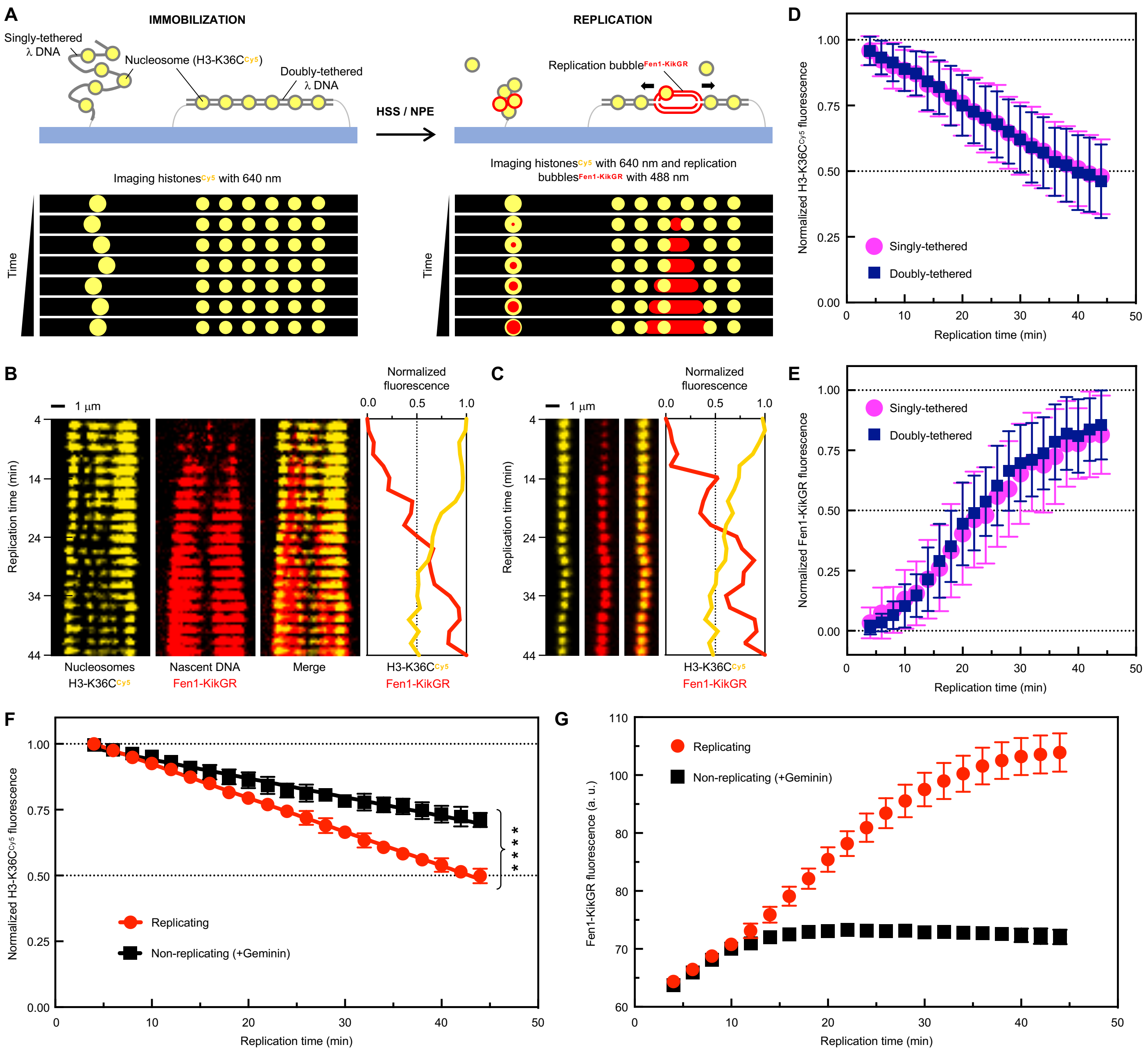

Fig. S4. Replication of singly- and doubly-tethered fluorescent λ nucleosomes in *Xenopus* egg extracts. (**A**) Schematic of the experimental set-up. Doubly- and singly-biotinylated λ DNA molecules containing fluorescent nucleosomes are attached to the surface so that approximately 50 % of the molecules are tethered in a stretched form while the remaining molecules are tethered at only one end. Doubly-tethered λ nucleosomes display the characteristic 'beads-on-a-string' appearance, whereas singly-tethered λ nucleosomes show up as a spot of fluorescence that can be stretched under buffer flow to reveal the individual 'beads' (movie S1). The immobilized DNA is licensed in high-speed supernatant (HSS), which causes the singly-tethered molecules to compact (they no longer stretch under flow) due to the deposition of extract proteins (Supplementary Text). Replication is initiated upon introduction of nucleoplasmic extract (NPE) supplemented with a fluorescent fusion protein Fen1-KikGR, which decorates replication bubbles. Firing was unrestricted in these experiments. Cy5-labelled histones and Fen1-KikGR are imaged with 640-nm and 488-nm laser, respectively. To minimize photobleaching, images were taken at two-minute intervals. Replication of singly-tethered λ nucleosomes is manifested by the appearance and subsequent increase of Fen1-KikGR fluorescence, colocalized with the H3-K36C^{Cy5} signal. (**B** and **C**) Kymograms and corresponding intensity profiles for doubly- (B) and singly-tethered (C) λ nucleosomes. Kymograms of H3-K36C^{Cy5} fluorescence (yellow; left panels), Fen1-KikGR (red; central panels) and both signals

together (merge; right panels) are presented. Time and size scales are indicated. (**D**) Plot showing the mean loss of H3-K36C^{Cy5} fluorescence for doubly- (blue squares) and singly-tethered (magenta circles) λ nucleosomes during replication under unrestricted firing conditions. 110 molecules were analyzed for each data set. Individual fluorescence decay traces were normalized to background ('0') and maximum values of fluorescence ('1'). A mean fluorescence value and standard deviation were calculated and plotted for each time point. We observed no difference in the loss of H3-K36C^{Cy5} fluorescence between the doubly- and singly-tethered λ nucleosomes. (**E**) Plot showing the mean increase of Fen1-KikGR fluorescence for doubly- (blue squares) and singly-tethered (magenta circles) λ nucleosomes during replication under unrestricted firing conditions. 110 molecules were analyzed for each data set. Individual fluorescence decay traces were normalized to minimum ('0') and maximum values of fluorescence ('1'). A mean fluorescence value and standard deviation were calculated and plotted for each time point. We observed no difference in the firing timing between the doubly- and singly-tethered λ nucleosomes. (**F**) Plot showing the mean loss of H3-K36C^{Cy5} fluorescence for 1:1 mixture of doubly- and singly-tethered λ nucleosomes during replication under unrestricted firing conditions (red circles) versus non-replicating control (black squares; +Geminin). 36 fields of view were analyzed for each data set. Decay traces for individual fields of view were normalized to background ('0') and maximum values of fluorescence ('1'). A mean fluorescence value and standard deviation were calculated and plotted for each time point. Data sets were fitted to a linear regression model (see table S2 for fitting parameters) and the statistical significance of the differences between the slopes was estimated as <0.0001. The loss of histone-associated fluorescence is significantly faster for replicating nucleosomes than for nucleosomes incubated in non-replicating extracts (see Supplementary Text for further details and panel G for the replication efficiency comparison). (**G**) Plot showing the mean increase of Fen1-KikGR fluorescence for 1:1 mixture of doubly- and singly-tethered λ nucleosomes during replication under unrestricted firing conditions (red circles) versus non-replicating control (black squares; +Geminin). 36 fields of view were analyzed for each data set. A mean fluorescence value and standard deviation were calculated and plotted for each time point.

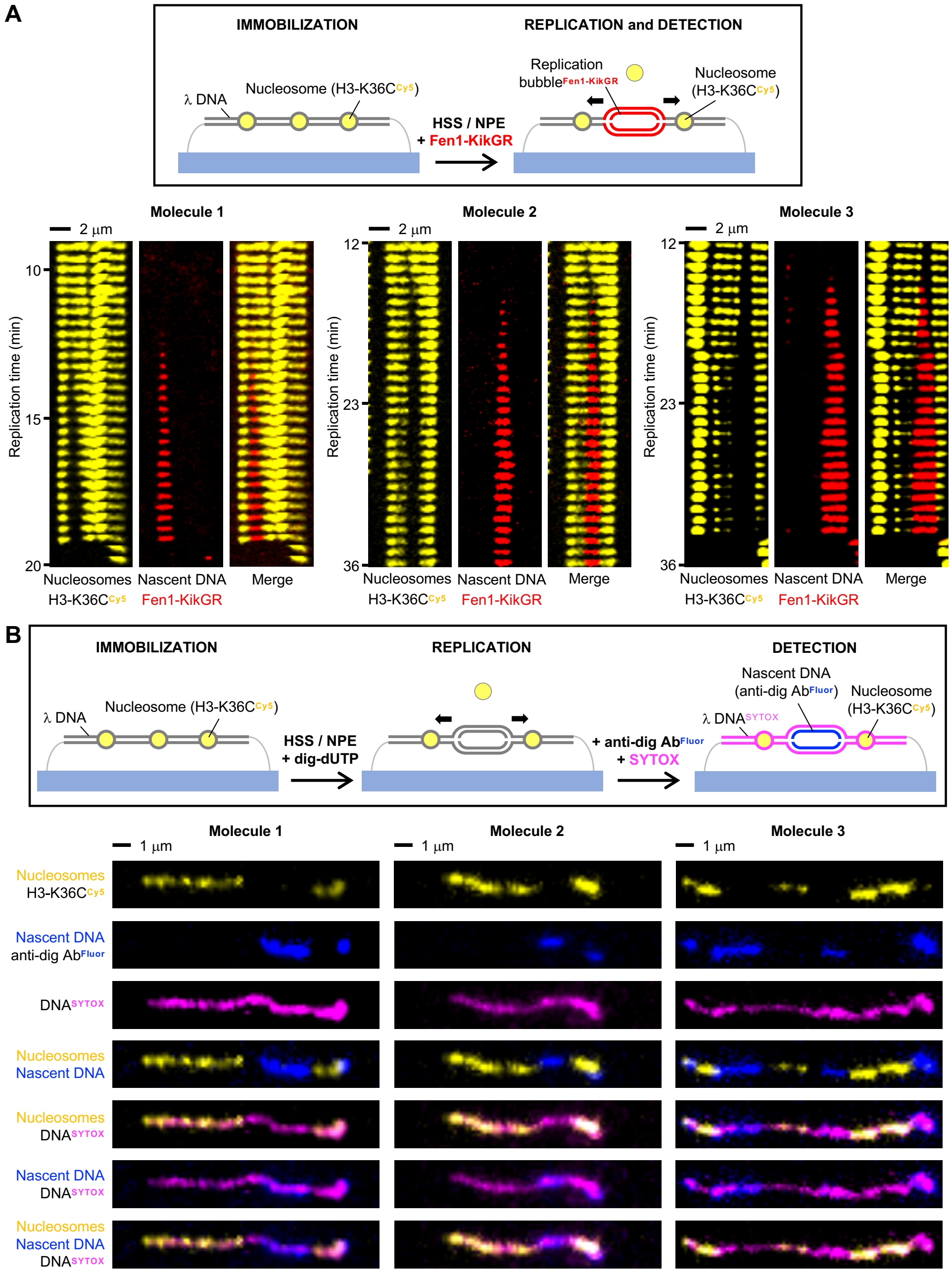

Fig. S5. Replication of doubly-tethered λ DNA molecules saturated with fluorescent nucleosomes in *Xenopus* egg extracts in the presence and absence of Fen1-KikGR. (**A**) Real-time imaging of λ nucleosomes replicated in extracts supplemented with Fen1-KikGR. Schematic of the experimental set-up is presented in black borders whereas the bottom panel shows real-time replication of three individual λ molecules. Data are presented as kymograms of nucleosome-associated fluorescence (yellow), Fen1-KikGR signal indicating nascent DNA (red) and both signals together (merge). Time and size scales are indicated. Fluorescent nucleosomes are predominantly evicted from the DNA templates upon the replication fork encounter (as manifested by the loss of histone-associated fluorescence) indicating that nucleosome density does not affect the efficiency of histone recycling. (**B**) Post-replication imaging of λ nucleosomes replicated in extracts supplemented with digoxigenin-11-dUTP (dig-dUTP), instead of Fen1-KikGR. Schematic of the experimental set-up is presented in black borders. The bottom panel shows post-replication detection for three individual λ molecules: H3-K36C^{Cy5} nucleosomes (yellow), nascent DNA (blue; anti-dig Ab^{Fluor}) and overall DNA (magenta; SYTOX Orange). Nucleosome-free zones colocalize with nascent DNA tracts, indicating that H3-K36C^{Cy5} histones do not efficiently transfer behind the replication fork in the absence of Fen1-KikGR. Note that the high salt wash, required for replication

termination in this assay, leads to non-specific sticking of the immobilized DNA molecules to the surface, which appear bent rather than straight lines under the microscope. Note that experiments presented in panels A and B were run in parallel using the same extract mixture, which was split and supplemented with either Fen1-KikGR for real-time replication visualization (A) or dig-dUTP for post-replication detection (B). After 30 minutes, replication was stopped by flowing in buffer containing 20 mM Tris pH 7.5, 10 mM EDTA and 0.5 M NaCl. Under these conditions, extracts were washed out from the flow cell but nucleosomes remained intact on the immobilized DNA and were imaged with 640-nm laser. Nascent DNA, containing dig-dUTP incorporated during replication, was visualized through immunostaining with an anti-digoxigenin antibody labelled with fluorescein (anti-dig Ab^{Fluor}; excited with 488-nm laser). In addition, non- and replicated DNA was stained with SYTOX Orange and visualized using 561-nm laser.

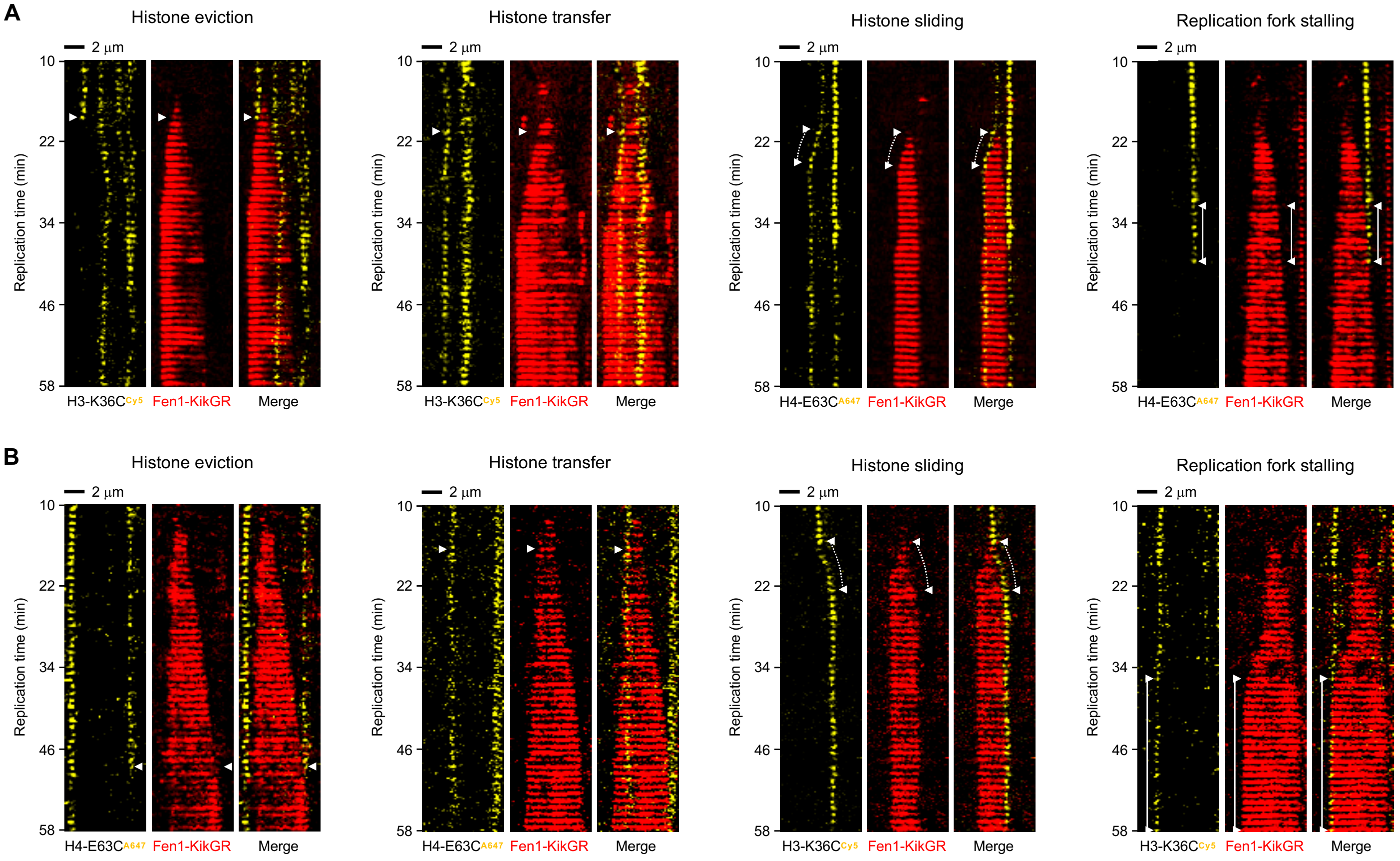

Fig. S6. Outcomes of the replication fork collision with nucleosomes during DNA replication in *Xenopus* egg extracts depleted of histones H3 and H4. For each specified outcome, data are presented as kymograms of nucleosome-associated fluorescence (H3-K36C^{Cy5} or H4-E63C^{A647}; yellow; left panels), Fen1-KikGR signal indicating nascent DNA (red; central panels) and both signals together (merge; right panels). Time and size scales are presented. The white triangles mark the point of initial encounter between the replication fork and nucleosome. Dotted lines indicate sliding events, whereas solid lines correspond to replication fork stalling. (**A**) Outcomes of the replication fork collision with nucleosomes during replication in *Xenopus* egg extracts depleted of histone H4 and H3 (ΔH4/H3). (**B**) Outcomes of the replication fork collision with nucleosomes during replication in *Xenopus* egg extracts depleted of endogenous histone H4 and H3, and supplemented with recombinant histones H4 and H3 (ΔH4/H3 + rH4/H3).

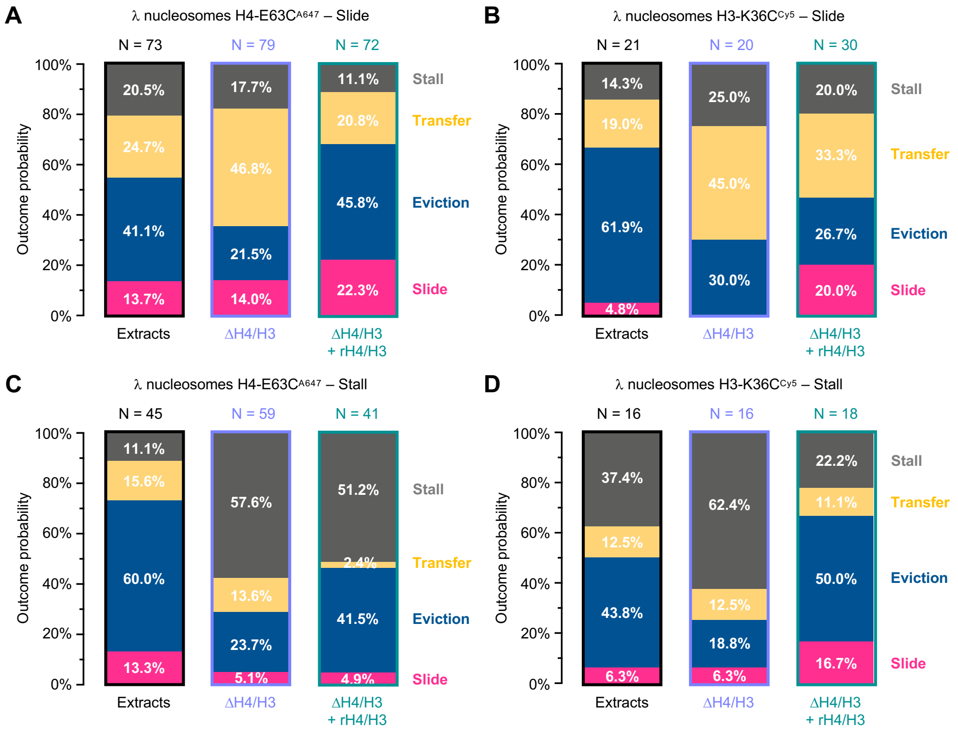

Fig. S7. Quantification of the secondary outcomes of replication fork collision with nucleosomes. Collisions were analyzed for assays conducted in regular undepleted extracts (black borders; left panels), extracts depleted of histones H4 and H3 (blue borders; central panels; ΔH4/H3) and extracts depleted of endogenous histones but supplemented with recombinant H4 and H3 (green borders; right panels; ΔH4/H3 + rH4/H3). N indicates the total number of analyzed collisions. For each condition, data from at least two biological repeats were pooled in the analysis. (**A**) Quantification of the outcome of histone sliding for λ nucleosomes containing H4-E63C^{A647}. (**B**) Quantification of the outcome of histone sliding for λ nucleosomes containing H3-K36C^{Cy5}. (**C**) Quantification of the outcome of replication fork stalling for λ nucleosomes containing H4-E63C^{A647}. (**D**) Quantification of the outcome of replication fork stalling for λ nucleosomes containing H3-K36C^{Cy5}.

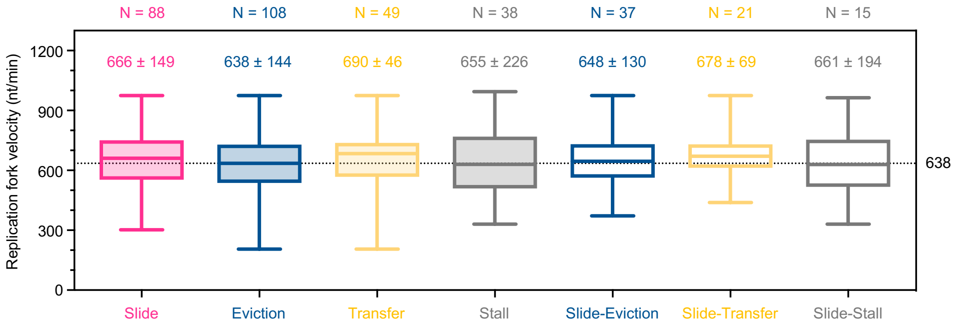

Fig. S8. Effect of replication fork velocity on the outcome of nucleosome-fork encounter. Box-and-whisker Tukey plot of replication fork velocities measured in regular extracts and categorized by the collision outcome. Data for λ nucleosomes containing H4-E63C^{A647} and H3-K36C^{Cy5} were pooled to generate this plot. Values above the box plots indicate the mean replication fork velocity extracted from the Gaussian fit, plus and minus standard deviation. The number of values analyzed per data set (N) is also shown. The horizontal dotted line marks the mean replication fork velocity in regular extracts, 638 nt/min.

Supplementary Tables

Table S1. Fitting parameters for histone dynamics data in HSS. Corresponds to Fig. 2, D and E. N indicates the number of individual decay traces used to generate plots of the mean loss of fluorescence. Mean loss of fluorescence data were fitted to a one phase decay model, where Y = (Y_0 – Plateau)*exp(-K*X) + Plateau. Y_0 is the Y value when X (time) is zero, K is the rate constant and $t_{0.5}$ is the half-life, calculated as ln(2)/K.

Parameter	H2A-K119C^{Cy5}	H2B-T112C^{A647}	H3-K36C^{Cy5}	H3-T80C^{A647}	H4-E63C^{A647}
N	127	128	122	133	132
Y_0	1.476 ± 0.022	1.580 ± 0.039	1.154 ± 0.011	1.232 ± 0.015	1.123 ± 0.018
Plateau	-0.023 ± 0.018	0.102 ± 0.012	-0.094 ± 0.089	0.012 ± 0.060	-0.008 ± 0.129
Amplitude	1.500 ± 0.012	1.478 ± 0.030	1.248 ± 0.079	1.220 ± 0.047	1.131 ± 0.112
K (s^{-1})	(2.417 ± 0.091) x 10^{-3}	(3.448 ± 0.138) x 10^{-3}	(0.894 ± 0.099) x 10^{-3}	(1.148 ± 0.104) x 10^{-3}	(0.935 ± 0.172) x 10^{-3}
$t_{0.5}$ (s)	286.7	201.0	775.0	603.8	741.2
R^2	0.7683	0.6161	0.7725	0.7168	0.5362

Table S2. Fitting parameters for the loss of histone fluorescence data for replicating and non-replicating λ nucleosomes. Corresponds to fig. S4F. Data were fitted to a linear regression model, where Y = Slope* X + Y-intercept. In this case, the slope indicates the rate constant of the histone fluorescence loss.

**Parameter**	**Replicating**	**Non-replicating**
Slope (min^{-1})	-0.01288 ± 0.00007	-0.007149 ± 0.00009
Y-intercept	1.052 ± 0.002	1.012 ± 0.002
X-intercept (min)	81.72	141.6
1/slope (min)	-77.66	-139.9
R^2	0.9787	0.9002

Supplementary Movies

Movie S1. Singly-tethered low-density λ nucleosomes containing H3-K36C^{Cy5} (yellow) imaged in buffer under 'no-flow' conditions followed by 50 µl/min flow. Without buffer flow low-density nucleosomes on a single λ DNA molecule appear as a diffraction-limited spot of fluorescence. The molecule unfolds and stretches under flow, unveiling nucleosomes distributed along λ as 'beads-on-a-string'. The scale bar corresponds to 2 µm. The movie is presented in real time and spans 35 sec (4.8 frames per second). Flow is started at 16 sec.

Movie S2. Doubly-tethered high-density λ nucleosomes containing H3-K36C^{Cy5} (yellow) imaged in buffer under 'no-flow' conditions followed by 50 µl/min flow. Doubly-tethered λ molecule saturated with nucleosomes does not stretch under flow, and thus appears as a diffraction-limited spot of fluorescence throughout the movie. The scale bar corresponds to 2 µm. The movie is presented in real time and spans 40 sec (4.8 frames per second). Flow is started at 20 sec.

Movie S3. Stretched λ nucleosomes containing H2A-K119C^{Cy5} (yellow) during incubation in HSS; corresponds to Fig. 2B. The movie covers 11 min of imaging during incubation in HSS (3-14 min). 1 sec of the movie corresponds to 2.8 min in real time. The scale bar corresponds to 2 µm.

Movie S4. Stretched λ nucleosomes containing H2B-T112C^{A647} (yellow) during incubation in HSS; corresponds to Fig. 2B. The movie covers 11 min of imaging during incubation in HSS (3-14 min). 1 sec of the movie corresponds to 2.8 min in real time. The scale bar corresponds to 2 µm.

Movie S5. Stretched λ nucleosomes containing H3-K36C^{Cy5} (yellow) during incubation in HSS; corresponds to Fig. 2C. The movie covers 11 min of imaging during incubation in HSS (3-14 min). 1 sec of the movie corresponds to 2.8 min in real time. The scale bar corresponds to 2 µm.

Movie S6. Stretched λ nucleosomes containing H3-T80C^{A647} (yellow) during incubation in HSS; corresponds to Fig. 2C. The movie covers 11 min of imaging during incubation in HSS (3-14 min). 1 sec of the movie corresponds to 2.8 min in real time. The scale bar corresponds to 2 µm.

Movie S7. Stretched λ nucleosomes containing H4-E63C^{A647} (yellow) during incubation in HSS; corresponds to Fig. 2C. The movie covers 11 min of imaging during incubation in HSS (3-14 min). 1 sec of the movie corresponds to 2.8 min in real time. The scale bar corresponds to 2 µm.

Movie S8. Example of nucleosome-fork collision resulting in histone eviction; corresponds to Fig. 3A. Histone eviction is manifested by the loss of histone fluorescence (H4-E63C^{A647}; yellow) at the point of collision with the progressing replication fork (Fen1-KikGR; red). The movie covers 36 minutes of imaging during replication (10-46 min). 1 sec of the movie corresponds to 10.3 min in real time. The scale bar corresponds to 2 µm.

Movie S9. Example of nucleosome-fork collision resulting in histone transfer behind the replication fork; corresponds to Fig. 3B. Histone transfer is observed when the histone-associated fluorescence H4-E63C^{A647}; yellow) is retained and incorporated into the track of replicated DNA (Fen1-KikGR; red). The movie covers 36 minutes of imaging during replication (10-46 min). 1 sec of the movie corresponds to 10.3 min in real time. The scale bar corresponds to 2 µm.

Movie S10. Example of nucleosome-fork collision resulting in histone sliding; corresponds to Fig. 3C. Histone sliding is observed when the histone-associated fluorescence (H4-E63C^{A647}; yellow) moves together with

the tip of the replication bubble (Fen1-KikGR; red). The movie covers 36 minutes of imaging during replication (10-46 min). 1 sec of the movie corresponds to 10.3 min in real time. The scale bar corresponds to 2 μm.

Movie S11. Example of nucleosome-fork collision resulting in replication fork stalling; corresponds to Fig. 3D. Replication fork stalling occurs when nucleosome constitutes a roadblock preventing the replication fork from further movement and is manifested by an arrested tip of the replication bubble (Fen1-KikGR; red) next to a static histone signal (H4-E63C^{A647}; yellow). The movie covers 36 minutes of imaging during replication (10-46 min). 1 sec of the movie corresponds to 10.3 min in real time. The scale bar corresponds to 2 μm.

Movie S12. Example of nucleosome-fork collision resulting in histone sliding followed by eviction; corresponds to Fig. 4A. H4-E63C^{A647} histones are shown in yellow and the Fen1-KikGR-decorated replication bubble is shown in red. The movie covers 36 minutes of imaging during replication (10-46 min). 1 sec of the movie corresponds to 10.3 min in real time. The scale bar corresponds to 2 μm.

Movie S13. Example of nucleosome-fork collision resulting in replication fork stalling followed by histone eviction; corresponds to Fig. 4B. H4-E63C^{A647} histones are shown in yellow and the Fen1-KikGR-decorated replication bubble is shown in red. The movie covers 52 minutes of imaging during replication (10-62 min). 1 sec of the movie corresponds to 10.3 min in real time. The scale bar corresponds to 2 μm.

Movie S14. Example of nucleosome-fork collision resulting in histone sliding followed by transfer; corresponds to Fig. 4C. H4-E63C^{A647} histones are shown in yellow and the Fen1-KikGR-decorated replication bubble is shown in red. The movie covers 36 minutes of imaging during replication (10-46 min). 1 sec of the movie corresponds to 10.3 min in real time. The scale bar corresponds to 2 μm.

Movie S15. Example of nucleosome-fork collision resulting in replication fork stalling followed by histone transfer; corresponds to Fig. 4D. H4-E63C^{A647} histones are shown in yellow and the Fen1-KikGR-decorated replication bubble is shown in red. The movie covers 36 minutes of imaging during replication (10-46 min). 1 sec of the movie corresponds to 10.3 min in real time. The scale bar corresponds to 2 μm.

Movie S16. Example of nucleosome-fork collision resulting in histone sliding followed by replication fork stalling; corresponds to Fig. 4E. H4-E63C^{A647} histones are shown in yellow and the Fen1-KikGR-decorated replication bubble is shown in red. The movie covers 36 minutes of imaging during replication (10-46 min). 1 sec of the movie corresponds to 10.3 min in real time. The scale bar corresponds to 2 μm.

Movie S17. Example of nucleosome-fork collision resulting in replication fork stalling followed by histone sliding; corresponds to Fig. 4F. H4-E63C^{A647} histones are shown in yellow and the Fen1-KikGR-decorated replication bubble is shown in red. The movie covers 36 minutes of imaging during replication (10-46 min). 1 sec of the movie corresponds to 10.3 min in real time. The scale bar corresponds to 2 μm.
